# Practices of patient engagement in drug development: a systematic scoping review

**DOI:** 10.1186/s40900-022-00364-8

**Published:** 2022-06-29

**Authors:** Olga Zvonareva, Constanța Craveț, Dawn P. Richards

**Affiliations:** 1 Department of Health, Ethics and Society, Maastricht University, Minderbroedersberg 4-6, 6211 LK Maastricht, The Netherlands; 2 Five02 Labs, Inc., Toronto, ON Canada

**Keywords:** Patient engagement, Patient participation, Drug development, Systematic scoping review

## Abstract

**Background:**

During the past decade, patient engagement (PE) has attracted significant attention in the field of drug development. Readiness to accept the central importance of patients’ knowledge and contributions has become evident. This study aimed to synthesize evidence on the current state of PE in drug development: what is actually being done and how.

**Methods:**

A systematic scoping review was conducted based on a PRISMA-informed protocol. Search was performed in PubMed, EMBASE and Web of Science, covering the period between 2011 and 2021. For analysis of extracted data, we developed a framework for analyzing PE in Drug Development. The Framework distinguishes a number of different PE types that take place at different stages of drug development and are characterized by the different degrees of power patients have in the process. It allowed us to assess depth and intensity of PE initiatives included in this review.

**Results:**

Most included PE initiatives took place at the stage of designing studies (40 in total). At this stage drug development goals are already set, but the mode of reaching them has not yet been fully determined. PE initiatives on the finetuning details stage followed (16 in total). The finetuning details stage covers the last parts of the drug development trajectory, when only relatively minor issues are still open for patients’ contributions. The least numerous were PE initiatives on the stage of setting up R&D program (13 in total). This stage refers to the early steps in drug development where PE has the potential to make the most impact on shaping the subsequent process. In terms of intensity of engagement, most PE initiatives included in this review align with consultation and involvement types, 26 and 30 initiatives, respectively. Partnership was less frequent in the published accounts of PE (13 initiatives).

**Conclusions:**

This review delineated a contemporary landscape of PE in drug development. Although attention to PE in drug development is relatively recent, a wide range of PE practices has already been initiated. The results indicate the necessity of distinguishing between different types of PE in order to understand consequences of choices regarding depth and intensity of PE.

**Supplementary Information:**

The online version contains supplementary material available at 10.1186/s40900-022-00364-8.

## Plain English summary

This article summarizes what is publicly found in scientific papers about patient engagement in drug development initiatives between 2011 and 2021. It also introduces a new Framework to use to look at these patient engagement efforts. The Framework breaks these efforts down based on the depth of patient engagement in the drug development process and different degrees of influence or power patients have or intensity of engagement. In terms of depth of patient engagement in the process of drug development, most patient engagement initiatives described efforts involved in designing studies where goals were already set. Next were patient engagement efforts related to finetuning details where patients could make minor contributions. The fewest efforts were found related to setting up a research and development program where patients potentially could make significant impact. In terms of intensity of patient engagement, most initiatives aligned with consultation and involvement intensities, and few examples aligned with the highest intensity of patient engagement that was considered partnership. While patient engagement in drug development is becoming more common, the approaches to doing so vary widely. We have developed a new Framework to help characterize these efforts related to patient influence in the process as well as depth of their engagement.

**Supplementary Information:**

The online version contains supplementary material available at 10.1186/s40900-022-00364-8.

## Background

During the past decade, attitudes towards patient engagement (PE) in drug development have changed significantly. Until recently, it was typical for regulators, industry, and academic researchers to think of patients mostly as clinical trial participants with their contribution limited to data provision. This view contrasted with those existing in other health-related spheres, such as healthcare priority-setting and services delivery, where patient and public engagement has been increasingly practiced for about thirty years [1, 2]. However, readiness to reconsider the role of patients, and acknowledge the central importance of their lived knowledge and contributions, is now becoming more evident in the drug development field as well [3].

Different stakeholders have articulated a range of expectations in connection to PE. Many of these expectations arise in the context of concerns over declining productivity of contemporary drug development. Drug development enterprise participants often mention rising costs, administrative delays, inefficiencies, and high failure rates among obstacles that, together, beset the progress [4, 5]. PE, consequently, is framed as a potential answer to these obstacles. By putting unmet health needs first, using outcomes relevant for patients, and designing trials to be more convenient for participation, drug developers expect to decrease the chances of costly late-stage failures and address wide-spread problems with recruitment and retention in clinical trials [6, 7].

Hand-in-hand with the expectations of improved productivity of the pharmaceutical industry, naturally emerge expectations of better quality and more relevant drugs [8]. Full consideration of patients’ priorities, experiences, and circumstances during the drug development process may deliver better solutions, as patients know best about what makes a meaningful difference to them. PE brings the question ‘what is needed?’ on a par with the question ‘what is possible?’, the latter question being a more traditional one for pharmaceutical innovating [9]. Furthermore, improvements in the regulatory process are also expected. During thematic forums, in officials’ statements, and in dedicated publications, PE is discussed as holding a promise to make the regulatory reviews more responsive to the patients and even to speed up drug approval. For example, it is anticipated that if a company relies on patient preferences when defining endpoints to be used in clinical trials, its case would be clearer and more convincing for the regulators [10].

Finally, the rising interest and declared commitment to PE introduces the possibility of democratizing drug development. Explicit discussions of drug development democratization have been limited. More pragmatic expectations outlined above feature more frequently in PE advocates’ statements. Yet, recognition of the patients’ right to shape treatment options available to them is implicitly present in the notion of PE itself. For a long time, decisions regarding problems to address, profiles of drugs to develop, and modes of assessing candidate drugs have been made by a restricted group of stakeholders. This group has consisted mostly of those involved with the industry and, to a lesser extent, regulation and academic research. However, the consequences of these decisions are so far-reaching, affecting the health and lives of so many people, that opening drug development up for wider participation appears to be imperative.

With discussions about PE in drug development intensifying, we witness the emergence of regulatory initiatives aimed at facilitating PE, as well as efforts to develop guidance for undertaking PE in practice [11, 12]. Scholarly attention has turned to exploring attitudes to PE among various stakeholders, ascertaining effects of particular PE instances, and developing tools for evaluating PE [13–15]. What has remained relatively less studied is the overall landscape of PE in drug development over the course of the recent decade: what is actually being done, where, and by whom. The study reported here aims to address this gap. Adding to the reviews that focused on specific fields, such as antimicrobial drug development [16], and on specific stages, such as preclinical laboratory research [17], this article offers a more general review of PE in drug development.

Since PE may take different forms and can be initiated at different stages of the drug development process, we developed a conceptual framework that reflects this diversity. This article draws on this new framework, described in the next section, to provide a meaningful snapshot of the contemporary state of PE in drug development. After describing the framework, we outline methods employed to conduct this review, followed by presentation of the results. The results section begins with a general picture of PE in drug development: when accounts of PE initiatives included in this review were published, where these initiatives were conducted, and by whom. Further, we delineate how the identified PE initiatives map into the different types of PE proposed by our framework and provide detailed descriptions of each type with illustrative examples.

### Framework for analyzing patient engagement in drug development

Existing definitions of PE vary considerably. Much of the conceptual work of defining PE has been done in the field of health care. For instance, Carman et al. [18] defined PE in health care as ‘patients, families, their representatives, and health professionals working in active partnership at various levels across the health care system—direct care, organizational design and governance, and policy-making—to improve health and health care’ (p. 224). To add complexity, some authors prefer to use the term involvement and/or also include public alongside patients, as for example Tritter [19] who defined patient and public involvement as ‘[w]ays in which patients can draw on their experience and members of the public can apply their priorities to the evaluation, development, organization and delivery of health services’ (p. 276).

Literature on PE in drug development has offered less conceptual input. Rather, it generally tends to emphasize partnership with patients and inclusion of patients’ voice across the entire cycle of medicines development. Several insights from the literature on PE in health care are particularly relevant for further conceptualizing PE in drug development. First, those who are engaged may be patients, but may also be caregivers and the general public [20]. Second, engagement may take place at different stages or levels of an activity in question. In the field of health care such levels could be, for example, direct care, organizational design and governance, and policy making [18]. Third, engagement may take different forms that can be positioned on a continuum from lower to higher degrees of patients’ power or influence and decision-making authority [21].

**Fig. 1:**
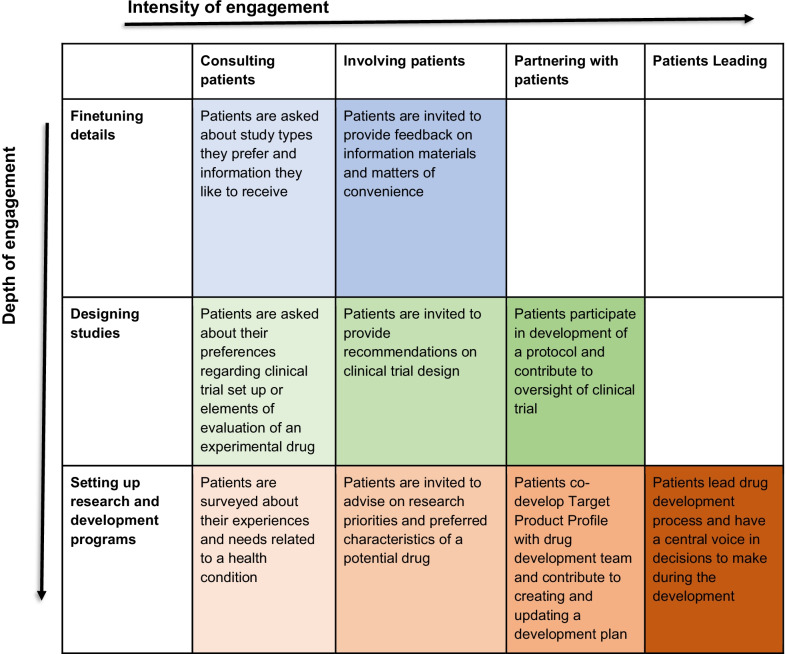
Framework for analyzing patient engagement in drug development

Taking account of these insights and, in particular, drawing on visualizations of engagement continuums by Carman et al. [18] and Spectrum of Public Participation by International Association for Public Participation [22], we developed a framework for analyzing PE in drug development (Fig. 1).

The first dimension of the Framework concerns the intensity of engagement. Here, different types of engagement are understood as forming a continuum. On the left side of the continuum, patients’ roles are less active and their participation in shaping agendas and decision-making is limited. On the right side of the continuum, patients are active partners in shaping agendas and making decisions and may have more power and responsibility than other stakeholders. This continuum can be broken down into a number of tentatively discreet types. The continuum in this Framework begins from *consultation*, which is understood as asking patients for their views to inform decisions in the drug development process, but without any obligation to act on these views. Then follows *involvement*, which is a dialogue or interaction with patients with a degree of mutual influence and accountability. Further, the continuum moves to *partnership*—active, ongoing and equal collaboration between drug developers and patients, both groups broadly conceived. Finally, the fourth type is *patient leadership*, when drug development is driven by patients who decide who else and when to invite.

Since the continuum spans from low to high intensity of engagement, it may be tempting to conclude that the higher the intensity the better. This conclusion could be valid in many situations, but in others lower intensity engagement may be appropriate either due to the nature of an issue at hand, type of a question to answer, or particularities of the situation itself. At the same time, it should be noted that movement from lower to higher intensity of engagement is associated with movement from one-off PE instances to more sustained and continuous collaboration. Partnerships, for instance, are more likely to be ongoing than consultations which tend to be arranged as isolated exercises. Therefore, it is possible to think of an engagement ecosystem where different types of engagement are practiced in connection to different purposes and issues and one-off PE instances take place alongside longer-term commitments.

The second dimension of the Framework focuses on the depth of engagement. Depth of engagement here is understood as being related to the drug development stage at which engagement is initiated. We distinguish three stages positioned from later stage to earlier stage engagement. The first stage is *finetuning details*, when patients engage in the drug development at the late stages, after all core decisions are already taken and only minor implementation issues are to be decided upon, for example, checking wording in trial informed consent forms or dissemination materials. The second stage is *designing studies*, when patients engage in the drug development process mid-way, when the mode of reaching the drug development goals has already been decided upon. Finally, the third stage is *setting up research and development (R&D) programs*, when patients engage in the drug development process (almost) from the beginning, at the point of delineating unmet needs and setting up the research agenda. Correspondingly, the earlier engagement takes place the more impact on drug development can be expected.

The Framework has empty cells. This is because the kinds of PE that would fit these cells are illogical and/or hard to conceive in practice. Partnership and, especially, patient leadership require initiation at the earlier stages of drug development because initiation at the later stages would mean that the most fundamental decisions have already been made. Consequently, patients would not be able to play a role of equal collaborators or leaders, implied by partnership and patient leadership types of engagement. For example, when a partnership with patients is sought to co-develop trial information materials or dissemination tools, it can be doubted whether such an initiative represents a partnership because patients participate in making decisions of comparatively minor importance in the overall drug development scheme. At the same time, initiation at the earlier stages does not preclude patients from subsequently engaging in activities falling under the finetuning details stage.

## Methods

### Study design

This systematic scoping review was conducted according to a protocol developed prior to the literature search. The Preferred Reporting Items for Systematic Reviews and Meta-Analyses (PRISMA) guided the reporting [23]. The review is termed systematic because of the systematic search and extraction of data; it is termed scoping because it aims at mapping PE initiatives and does not involve quality appraisal of the included articles. Definitions of types of PE employed in this study follow the framework for analyzing Patient Engagement in Drug Development, described above.

### Data sources and search strategy

An electronic search strategy was developed by a trained search strategist and adapted for the following databases: EMBASE, PubMed and Web of Science. The search strategy included a combination of medical subject headings (MeSH) terms such as ‘patient participation’ and ‘drug development’ and words/phrases related to patient engagement at different stages of drug development (see Additional file 1: for summary of search strategies). Search terms were derived from a prior background literature search, tested and updated based on the test results and taking into account suggestions by the databases’ search engines. Other similar relevant terms were found in the literature or suggested by the databases’ search engines. The search specifically focused on the “patient” as a “research partner” engaged in shaping drug research and development. Background literature search indicated that the terms “consumer” or “public” participation, while prominent in many fields, were rarely used specifically in the field of drug development. This terminological particularity emerged during the initial shaping of the field of PE in drug development and reflects wording choices made by widely read authors. Scholars who cited their works subsequently tended to adhere to these choices. Therefore, the terms “consumer” and “public” participation were excluded due to their different theoretical and contextual meaning.

The search of the three databases was conducted on the 14th of April 2021. Furthermore, reference lists of the included studies were manually reviewed to ensure comprehensiveness. References were exported to a reference management software program (Zotero) and saved into the project library within Zotero.

### Eligibility criteria

We included peer-reviewed publications that reported initiatives to engage adult patients in any form of activities during any of the stages of drug research and development. All original publications were eligible if the initiatives reported implied some degree of an impact on drug development practice and provided sufficient detail on the process of PE. Thus, publications that reported studies of patients’ perspectives on aspects of drug development with no clear route to use these perspectives in shaping practice were excluded along with the publications which gave only minor details on how exactly PE was done. Only articles in English published between 2011 and 2021 were considered. The choice of a period is because PE in drug development is a relatively recent phenomenon. Background literature search suggests that widespread interest to PE has emerged during this decade. We did not include unpublished data or abstract-only articles. All commentaries and editorials were excluded, as well as reviews after their reference lists were manually checked.

### Study selection

Following the step of de-duplication, CC screened titles and abstracts of identified articles. As the screening was performed by a single team member, it introduced possibility of omissions. Articles that appeared to engage patients in drug development were retained and uploaded to Zotero (see Additional File 2: for a Flow Diagram of identified, screened and eligible publications). Then two reviewers, CC and OZ, independently screened retained articles based on the full text. Screened articles were classified by the reviewers into three categories: ‘Relevant’, ‘Possibly Relevant’, and ‘Irrelevant’. The resulting classifications were compared, differences discussed and reconciled, and the category ‘Possibly Relevant’ sorted into ‘Relevant’ and ‘Irrelevant’ categories. Articles agreed upon to belong to the category ‘Relevant’ were deemed eligible for further data extraction and analysis. All excluded articles were kept in a separate folder within the project Zotero library and reasons for exclusion were documented in an Excel spreadsheet for ease of monitoring and reporting.

### Data extraction

A data extraction form was developed to facilitate a systematic and transparent examination of included publications. The form was piloted and refined to ensure suitability for reaching the objectives of the review. The extracted characteristics were grouped in four clusters (see Additional file 3: Data extraction spreadsheet). The first cluster focused on the publication itself and included authors, their affiliations, and year of publication. The second cluster focused on the drug development activity where PE was implemented and included country, funding source, disease area, aim, study design, drug being developed, and population. The latter characteristic, population, was relevant for clinical trials primarily. The third cluster focused on PE and included data on who is engaged, how PE was initiated, PE methods, depth of engagement and intensity of engagement. Depth and intensity of engagement were judged based on the Framework for Analyzing Patient Engagement in Drug Development. Reasons for classifying instances of PE as belonging to a particular type were documented in the data extraction form. The fourth cluster included PE outcomes and, where reported, strengths and limitations of specific PE initiatives.

Data extraction began with both reviewers, CC and OZ, randomly selecting five articles found eligible for this review and independently extracting data from them according to the described standardized form. Afterwards, the extraction results were compared and any uncertainties regarding the extraction process were clarified. Further, CC proceeded to extract the data from remaining studies and OZ randomly checked data extraction for 25% of the included publications.

### Data analysis

The Framework for Analyzing Patient Engagement in Drug Development guided data analysis. It allowed to, first, organize included publications into groups according to depth and intensity of PE activities reported. Further, we examined methods and aims of PE activities within each group and compared the identified characteristics within and between the groups, producing a narrative synthesis of the data. Identified patterns enabled us to develop a map of the overall landscape of PE over the course of the recent decade and also to further specify the Framework employed in this review. We also developed case descriptions based on selected examples of different types of PE to illustrate similarities and differences between them.

### Quality assessment

Formal quality assessment criteria were not used in this review. Because the overall aim of this review was to characterize the landscape of PE in drug development in terms of PE types employed, we only ensured that included publications provided sufficient details on who was engaged, how, and for which purpose. The full list of included publications can be found in Additional File 4.

## Results

### PE in drug development: when, where, why and who

**Fig. 2:**
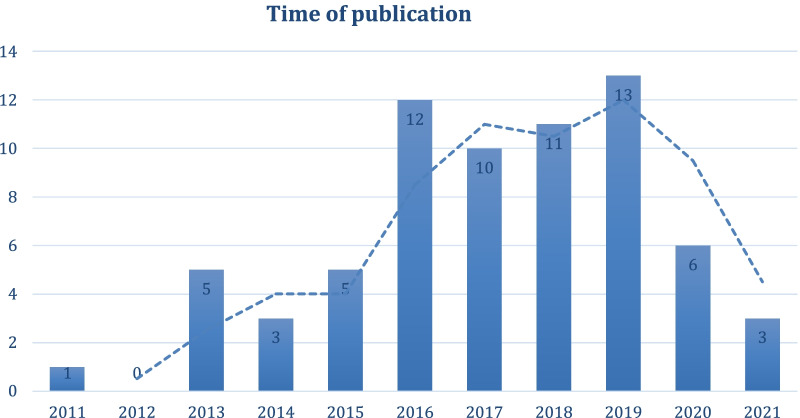
Number of publications per year (2011–2021)

In total, 69 publications were included. Most of the articles on PE in drug development included in this review were published in 2016–2019 (see Fig. 2 and Table 1). The rise in published accounts of PE by 2016 may have been stimulated by increasing attention of regulators and others to patients’ perspectives in the context of drug development and evaluation. For example, in 2012 the U.S. Food and Drug Administration (FDA) launched its Patient-Focused Drug Development (PFDD) initiative to understand patients’ experiences in specific disease areas and their views on currently available treatments [24]. In the European Union, 2012 became the year The European Patients’ Academy on Therapeutic Innovation (EUPATI) was launched by the Innovative Medicines Initiative, which is a public–private partnership [25]. A sharp decline in publications reporting on PE following 2019 can, arguably, be attributed to the COVID-19 pandemic, which made the publishing process more lengthy, complicated implementation of PE in practice, and could also have temporarily changed the priorities of drug developers, shifting attention away from PE.

**Table 1:** Geographical distribution of PE activities (note that each PE initiative reported in an article included in this review may have been conducted in more than one location; hence the number of locations is higher than the number of reported initiatives)

Number of publications	Country
47	USA
18	The UK
13	Germany
7	Australia, Canada, and Poland
6	Italy
5	Czechia and South Korea
4	Bulgaria Mexico, Norway and Spain
3	Brazil, France, Ukraine and Taiwan
2	Russia, China, Japan, Thailand, Slovakia, Sweden, Philippines, Ireland, Israel, India, Greece, Dominican Republic, Croatia, Colombia, and Chile
1	Estonia, South Africa, Romania, Puerto Rico, Malaysia, Hungary, Finland, Denmark, Cyprus, Costa Rica, Bosnia and Herzegovina, Japan, Austria, and Argentina
*	Europe = 6; North America = 1; South America = 1

**Fig. 3:**
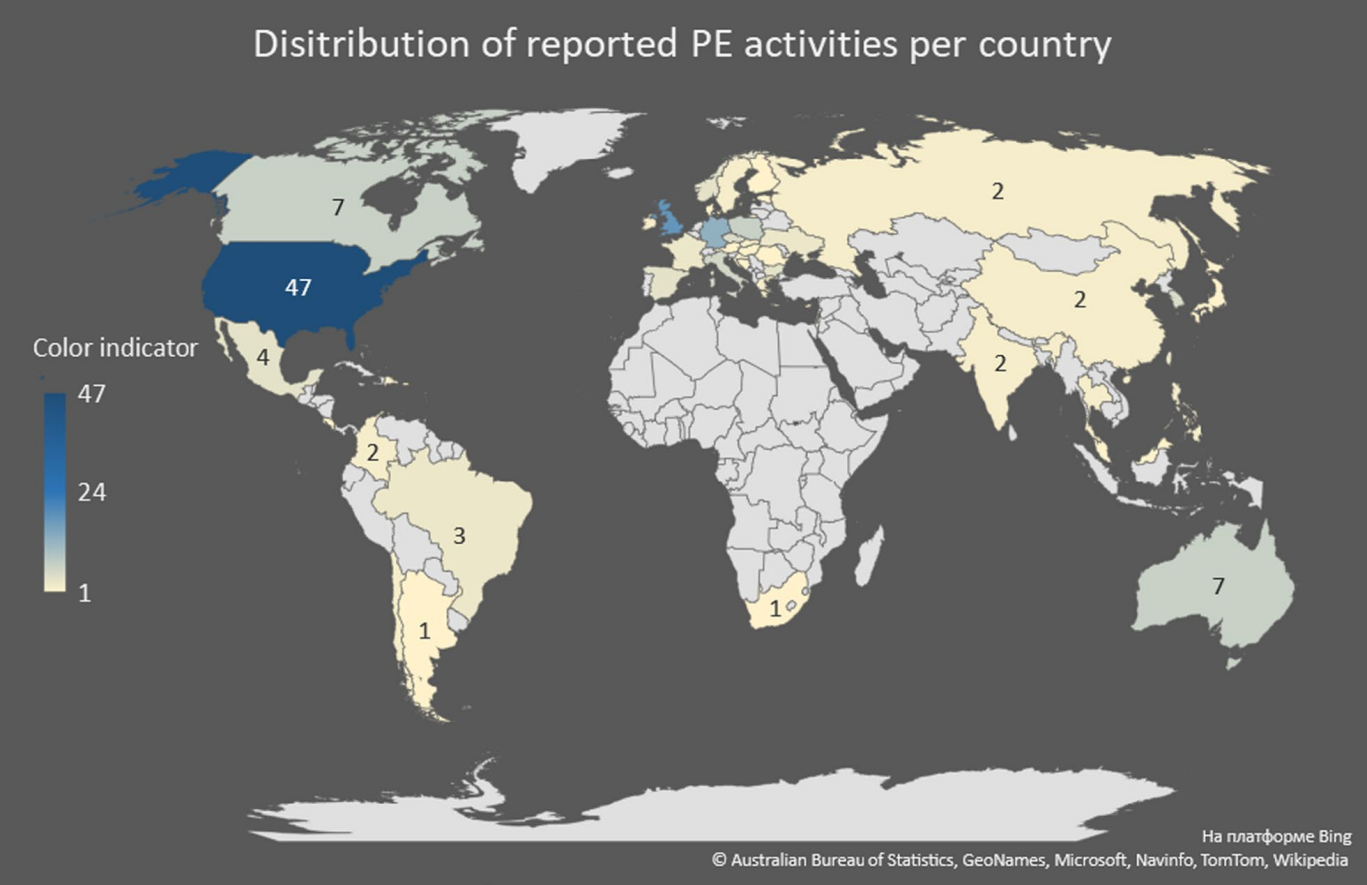
Geographical distribution of PE activities

The geographical distribution of PE activities reported in the articles included in this review is highly uneven (see Fig. 3, Table 1). Most of the reported activities took place in the U.S. (47). The U.K. and Germany are the two countries that follow the U.S. but with significantly less instances of PE, 18 and 13, respectively. Australia, Canada, Italy, and Poland account for 6 to 7 PE activities each. Notably, no PE in drug development was reported in countries in Africa, except for 1 instance in South Africa, and very little reported in countries in Asia and South America. Near absence of the countries that constitute what has been termed the Global South on the map of PE in drug development is surprising. Given a wealth of existing publications that report on efforts to make clinical trials more adapted and responsive to local perspectives in these settings [26, 27], some contribution into PE in drug development could well be expected. However, while expansion of clinical trial conduct into lower-income locations has been accompanied by efforts to engage local communities into trial planning and oversight, our review found little published evidence of PE in drug development.

There appear to be at least two complementary explanations for this picture. First, there is a tendency in academic publications to speak of community engagement when it comes to lower-income locations and of patient engagement or patient and public engagement when it comes to higher-income locations. While engagement initiatives may well configure communities as consisting of people who share a particular diagnosis, in practice community engagement usually concerns individuals who inhabit a particular place. Thus, engagement initiatives in the Global South tend to avoid engaging patients as such but rather focus more generally on local communities where clinical trials are conducted.

Second, clinical trials are undertaken not only in the context of drug development, but also for the purposes of testing public health interventions and non-pharmaceutical treatments. It is possible that engagement initiatives in lower-income locations tend to be associated with non-pharmaceutical trials, while pharmaceutical trials designed elsewhere are highly standardized by the time they land in these locations and have little space left for PE. Furthermore, PE at the stages of setting up R&D programs and designing studies that precede trial conduct, may be logistically challenging to conduct outside of higher-income locations, such as the U.S., where most drug development projects are currently initiated. Also, PE in lower-income locations may be further deprioritized since large pharmaceutical companies—the main players in the drug development arena, have devoted limited attention to developing drugs for diseases affecting the global poor, who disproportionately reside in these lower-income locations.

This latter point is illustrated by an overview of disease areas in which PE activities took place (see Table 2). Most PE initiatives reported in the articles included in this review took place within the context of developing drugs for non-communicable diseases. The cluster of infectious and parasitic diseases that take a heavy toll on populations residing in lower-income locations is represented by only 7 instances of PE in drug development for HIV/AIDS, Hepatitis C Virus, and COVID-19 pneumonia. This is not to suggest that drugs for non-communicable diseases are not relevant for people residing outside of the high-income countries. Rather we are highlighting that PE appears to occur more often when drugs are developed for non-communicable diseases compared to when drugs are developed for communicable diseases, perhaps, in part due to less drug development efforts devoted to communicable diseases more generally.

**Table 2:** Disease areas in which PE activities were reported

Disease areas	Nr. of publications
Non-communicable diseases	
Cancer	30
Autoimmune diseases	16
Rare diseases	10
Neurological and psychiatric conditions	8
Lung and esophageal disorders	7
Endocrine diseases	6
Cardiovascular diseases (CVD)	4
Chronic kidney disease (CKD)	1
Non-specific low back pain (NSLBP)	1
Communicable diseases	
Infectious diseases	7
HIV/AIDS	4
Hepatitis C Virus (HCV)	2
Severe or critical COVID-19 pneumonia	1

It was not always easy to clearly discern the initiators of PE in drug development. One relevant indication is a source of funding. From the information provided in the included publications we gathered that 3 initiatives were funded by charities, 3 initiatives by consortiums of public and private organizations, 23 initiatives by the pharmaceutical industry, and 42 initiatives by academic research funders and other public bodies. This latter group is rather diverse and includes such funders as the U.S. National Institutes of Health, universities, and various national research programs. There are situations when those who are initiating PE are different from those who are funding it. In cases of pharmaceutical companies, the funder and initiator are usually one entity. But in other cases, arrangements can be more complicated. For example, PE funded by an academic research funder could be conceived and initiated by a hospital, research association or a collaborative network that may include diverse actors such as patient advocacy organizations, state agencies, businesses, and others.

A trend noted in the process of analysis was a lack of financial involvement of industry at the stage of finetuning details in included publications. PE initiatives at this stage were funded and initiated by non-industry organizations, possibly due to their inability to reach earlier stages of drug development that tend to be carried out by industry. It is also possible that these initiatives were simply not reported by industry in the academic journals. The composition of organizations active at other stages is mixed.

### Diversity of PE activities: an overview

Among all PE initiatives described in the articles included in this review (see Table 3) most numerous are the ones taking place at the stage of designing studies (40 in total). At this stage drug development goals are already set, but the mode of reaching them has not yet been (fully) determined. In the PE initiatives classified in this review as belonging to this stage, patients were involved in deciding upon outcomes to consider, recommended amendments in trial design and organization primarily to improve enrollment and retention, contributed to development of tools for trial oversight, evaluated reporting process, and brought attention to ethical issues such as access to a drug being evaluated.

**Table 3:**
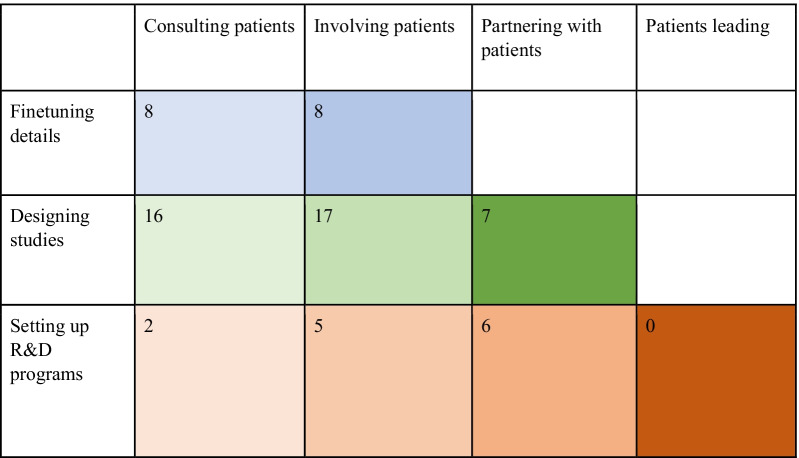
Types of PE based on the framework developed among the initiatives included in this review

Following PE initiatives in the designing studies stage, are initiatives on the finetuning details stage (16 in total). The finetuning details stage covers the last parts of the drug development trajectory, when only relatively minor issues are still open for patients to contribute to. Here patients played roles in informing patient recruitment strategies and evaluating and contributing to the development of information and education materials, informed consent forms, and such tools as decision aids.

Finally, the least numerous were PE initiatives on the stage of setting up R&D program (13 in total). This stage refers to the early steps in drug development where PE has the potential to make the most impact on shaping the subsequent process. In the initiatives that fell under this category patients participated in defining research agendas and priorities, choosing specific directions of inquiry to pursue and identifying barriers, provided insight into preferred outcomes and drug characteristics in terms of balance between risks and benefits, and contributed to designing drug development programs.

In terms of intensity of engagement, most PE initiatives included in this review align with consultation and involvement types, 26 and 30 initiatives, respectively. Both of these types imply a certain degree of preliminary framing by drug developers. That is, patients are invited to answer pre-formulated questions, discuss pre-formulated issues, and/or contribute to reaching pre-formulated goals. However, during a consultation drug developers and patients are at greater distance from each other and assume more clearly defined roles of those who ask questions and those who answer them. Involvement implies a more diverse spectrum of interactions and closer relations between the parties.

Partnership offers patients more opportunities to shape the space and conditions of their engagement. Parties are on a more equal footing and much less differentiation is apparent between those who engage and those who are engaged. This type of PE was less frequent in the published accounts of PE (13 initiatives), which could stem from a variety of reasons, including organizational complexity of setting up a partnership, uncertainty and resource requirements of this PE type, and simple underreporting in academic journals. The latter reason is very likely to partly account for zero initiatives of the patient leadership type included in this review. There are patient organizations that fund research related to the conditions of their focus. It is likely some of them also lead drug development efforts of their disease areas of interest but choose not to invest resources into writing articles about this experience for academic journals.

### PE at the stage of setting up an R&D program

At the stage of setting up an R&D program, the fewest initiatives followed the format of consultation (2 initiatives). It appears to be more typical for early-stage PE to involve more multidirectional communication and longer-term contacts than the consultation format allows. When the consultation format is selected, it is conceived as collecting data from the patients to inform drug development strategy and product design. Such data collection can be aided by digital technologies and online platforms, as an example demonstrates from an industry early drug development project for chronic obstructive pulmonary disease (COPD) [28]. In this example the company set up three studies. The first was a social media listening study—an analysis of online conversations in open-access platforms among patients with COPD conducted in the patients’ own words and without influence of researchers. The second was an online bulletin board exercise, where twenty COPD patients answered predefined questions derived from the previous study. Patients provided their answers asynchronously, in the course of two weeks within a moderated, closed online community platform, similar to a private chat room. The third was a patient preference study, where findings from the two previous studies were quantitatively evaluated via an online survey among patients with COPD. The authors of the publication where these studies were presented suggest that ‘collectively these patient insights and preferences will help assemble hypothetical treatment profiles with specific characteristics and also aid in selecting clinical outcome assessments beyond conventional end points in the COPD drug development program’ (p. 22).

Involvement and partnership are almost equally present at the stage of setting up R&D programs, 5 and 6 initiatives, respectively. Involvement practices tend to differ from pure consultations by a greater degree of dialogue and a possibility for patients to exert influence beyond simply allowing their experiences and preferences to be collected as data. An interesting example illustrating this point is an initiative by a regulatory agency [29]. On the one hand, the initiative did concern eliciting individual patient preferences, in this case, with regards to treatments for advanced melanoma and multiple myeloma. On the other hand, the instrument for eliciting preferences was developed in cooperation with two patient organizations, in which patients provided feedback on the technical aspects, content, and methodology, and the publication reporting on the process was written together by regulators and patients. The resulting patient preference elicitation methodology, applied at the stages preceding regulatory review early enough to ensure correspondence between a drug's characteristics and patients’ perspectives, may produce evidence to be included in marketing authorization applications. According to the authors of the publication, such information could provide support for ‘a claim of a favorable benefit–risk and inform the regulators’ decisions in situations where the balance of benefits and risks is not self-evident’ (p. 551).

Finally, partnership as a type of PE in drug development takes place in a more sustained manner. Conditions of partnership are not as pre-set as in the case of involvement and, especially, consultation, and what exactly the partnership is going to focus its activities on is defined jointly. An article, included in this review, describes an example of a partnership initiated by a pharmaceutical company, where the Patient Advocate Advisory Council (PAAC) was established [30]. The PAAC worked with the company representatives to design and execute a program whereby patients join clinical development teams. In parallel with developing a framework for patients and clinical development teams to work together, the PAAC conducted a pilot where, under a confidentiality agreement, a cancer patient advisor engaged with one of the clinical development teams, meeting key members of the team and providing feedback on the protocol and development program. While initiated by a pharmaceutical company, relationships between the PAAC and the company can be considered a partnership because PAAC members had a space to define different elements of the program themselves and try them out. A result of this partnership was characterized positively: it was agreed to expand the pilot to reach other development programs within the company and initiate engagement between patient advisors and development programs ‘even earlier than was possible with the pilot study. … despite the risk that therapy development programs in the earliest stages may not advance to later development’ (p. 350).

### PE at the stage of designing studies

Consultation initiatives were common at the stage of designing studies (16 initiatives). Those involved in designing studies formulate questions they would like to have information on and seek answers to these questions from the patients. Seeking answers in the cases of consultation may take a variety of forms, mostly quite restrictive. For example, a group of trialists from an academic hospital and with some industry affiliations developed a financial assistance program for cancer clinical trial participants to improve trial enrollment and retention [31]. Patients contributed to evaluation of this program by reporting their financial concerns and barriers to participation via survey. As a result of this evaluation the financial assistance program was considered effective and suitable for being implemented as a part of future trials. Another example, similarly illustrative of the boundaries pre-set for the patient input in the consultation format, is a formative study conducted by a non-profit organization to improve recruitment in its trials [32]. In this initiative people living with HIV/AIDS (PLWHA) diagnosed with cancer and invited to participate in a trial were offered to complete a survey about factors influencing their decision-making regarding trial participation and asked for recommendations about how to improve the organization’s trial accrual. Further, as typical for the consultation format, it was up to those asking questions, in this case trialists, to decide what to do with the input received. Thus, authors of the study that reported the initiative, concluded: ‘These suggestions present opportunities to the [organization] and its participating sites to consider ways to improve the appeal and experience of clinical trial participation and streamline the accrual process’ (p. 6).

Similar to consultation, involvement at the stage of designing studies constitutes a common type of PE (17 initiatives). One of the primary differences between the two types is the degree of mutual influence. During a consultation, patients rarely have an opportunity to go beyond the framework that predefines their role, questions posed, and the format answers should follow. In the case of involvement, the degree of freedom patients have to shape their input is higher. An example to illustrate this point comes from a study of tocilizumab for treatment of COVID-19 pneumonia conducted by an academic consortium [33]. While PE was not originally foreseen, a single-arm design of this trial was a result of a media campaign for giving a drug to all participating patients, instead of dividing them into experimental and control groups and giving a drug only to those in the experimental group. The campaign was spearheaded by physicians with support and under pressure from patients and led to a significant change in the study design in an attempt by investigators to strike the balance between scientific considerations and demands from physicians and patients. This situation suggests that involvement may not only be architectured by those designing studies but may well be uninvited, that is initiated by patients and their allies. Descriptions of invited involvement are more numerous in the publications included in this review. But in the cases of invited involvement at the stage of designing studies, patients’ influence is still broader than in the cases of consultation. This difference is noticeable in the PE initiatives that involve development of patient-reported outcomes (PROs)—tools for measuring outcomes that matter to patients [34]. Since these tools are meant to reflect patients’ perspectives, PE is necessary for PRO development. For example, creation of a novel PRO to evaluate therapy that is being developed for pantothenate kinase-associated neurodegeneration included interviews with professionals, patient advocates, and caregivers to inform the first version of the PRO [35]. This version was then piloted, finalized after patients who participated in piloting and primary caregivers provided their feedback on the first version during interviews, and used in a phase III trial.

Partnership as a form of PE at the stage of designing studies is seen less often (7 initiatives) and includes more prolonged engagement than consultation and involvement. Not only does it take time to set up a partnership, but the process of engagement itself in this case is inevitably lengthier because it is less scripted, more unpredictable, and cannot be limited to an isolated instance of feedback provision. For example, in an effort to facilitate clinical trials for facioscapulohumeral muscular dystrophy (FSHD) treatments, FSHD researchers initiated a series of meetings with industry and patients [36]. These meetings allowed identifying gaps in clinical trial readiness. In order to address these gaps, on the basis of FSHD Clinical Trial Research Network a study was developed to identify novel clinical outcome assessments and refine eligibility criteria for future clinical trials. The protocol for this study was informed by discussions between FSHD researchers, industry and patients and included provisions for ‘continuing dialogue throughout the course of the study’ (p.3). Continuing dialogue beyond development of the protocol itself was meant to ‘address specific aims or difficulties encountered in running the proposed study; for example, defining what would be clinically meaningful to people with FSHD, addressing concerns related to participating in clinical studies, and issues with recruitment and retention’ (p. 4). In this and other instances of partnership more prolonged engagement is likely to produce more transparency and, consequently, trust: remaining in touch with a particular project, patients also see what happened to their input.

### PE at the stage of finetuning details

PE at the stage of finetuning details focuses mostly on trial information materials and recruitment strategies. The analytical framework employed in this review distinguishes two types of PE at this stage: consultation (8 initiatives) and involvement (8 initiatives). The framework does not foresee the possibility of a partnership at this stage, because for a PE initiative to be a partnership it needs to be sufficiently prolonged for establishment of collaborative relationships and sufficiently deep to exert an impact beyond relatively minor aspects. This is not to suggest that partnerships cannot be concerned with information materials and recruitment strategies. Partnerships may well extend to include these items but are unlikely to focus exclusively on them.

Examples of PE at the stage of finetuning details found in the reviewed literature are quite similar and, indeed, do not resemble a partnership. Patients are invited to test information materials and tools and are asked about their experiences and expectations with regards to trial participation to facilitate recruitment and retention in trials. Consultation and involvement at this stage can be differentiated by looking at how pre-structured the patients’ input is. Consultation is more restrictive in this regard than involvement. For example, one consultation initiative carried out by an academic group aimed to test seven different strategies for recruitment of cancer patients and their caregivers in a randomized controlled trial [37]. After patients and their caregivers were contacted and invited to participate in this initiative, using seven strategies being tested, recruitment outcomes were compared. This initiative concluded that opt-out recruitment techniques are the most effective, yielding the highest number of participants, and should be used in future trial recruitment. This example illustrates how consultation initiatives at the stage of finetuning details channel patient input narrowly, not leaving opportunities for an unforeseen, patient-initiated feedback. In this case, patients and their caregivers could only indicate whether they would agree to participate in a study being contacted via a particular strategy.

Involvement at the stage of finetuning details is not drastically different from consultation, in part due to this stage itself limiting the scope of PE possibilities. When patients are involved, though, they have somewhat more space to articulate their views. For example, one involvement initiative by an academic group aimed to identify patients’ physical and psychosocial experiences of an investigational long-acting injectable pre-exposure prophylaxis (PrEP) product to aid in the development of patient and provider education materials [38]. Here patients were asked to rate their pain during and after injection on a five-point scale. This request is a closed one, similar to the request to make a choice whether or not to agree to study participation in the previous example. But the involvement example also included interviews with open-ended questions, where patients could direct a conversation and bring up issues investigators had not considered.

## Discussion and conclusion

This review delineated a contemporary landscape of PE in drug development. Although attention to PE in drug development is a relatively recent phenomena, a wide range of PE practices has already been initiated. These practices take place at varying stages of drug development and are characterized by different intensity of engagement. Using our novel Framework for analyzing PE in drug development, we were able to show that most reported PE initiatives took the form of consultation and involvement and occur at the stage of designing studies. Instances of partnership are fewer. Notable is the absence of reports about the patient leadership initiatives in the available academic literature .

The results indicate the necessity of distinguishing between different types of PE in drug development. While emergent scholarship and guidance documents tend to speak of PE in drug development as a relatively homogenous group of activities, this review indicates that in practice PE takes a wide variety of forms. Attention to this variety allows to elicit distinct positions accorded to or assumed by patients within engagement initiatives and different assumptions regarding the value and content of patients’ input embedded in the setup of specific PE practices. Importantly, distinguishing between different types of PE in drug development makes visible the consequences of choices regarding depth and intensity of PE. These consequences concern the impact patients are actually able to make on the drug development and the degree to which aspirations to take the patients’ voices seriously have been realized. Recognizing differences between PE types does not mean an obligation to strive for uniformly early and intense engagement in all situations. Rather, such recognition could facilitate building a PE ecosystem where different types of PE co-exist complementing each other.

The reported rise in diverse PE initiatives has been taking place against the backdrop of extensively articulated expectations regarding the capacity of patients’ input to cure drug development of its present-day maladies responsible for declining productivity. While evaluation of the PE outcomes was not the purpose of this review, it is hard to avoid discussing, however briefly, the significance of hopes pinned on PE in drug development for the future of PE. While being very far-reaching, expectations proposed by the existing literature are rather pragmatic: with patients’ input drug developers would be developing more relevant products, face less late-stage failures, experience less difficulties with trial recruitment and retention, and even have their products approved faster. These pragmatic expectations are, to a large extent, reflected in the empirical reports of PE initiatives included in this review. Not all reports provided information on the outcomes of PE, but those that did, focused on pragmatic outcomes such as satisfaction of patients with trial participation or improved relevance of end points.

These pragmatic outcomes are, without any doubt, of paramount importance. However, as mentioned in the introduction to this article, improving productivity is not the only rationale for PE in drug development. Of at least equal importance is the democratization rationale. Democratization rationale entails that since drug development priorities and practices affect lives and wellbeing of (almost) everyone, decisions in this domain must be opened up for wider participation. Yet, there is little explicit mention of democratization in the literature on PE in drug development and the reviewed empirical reports of PE initiatives do not evaluate the outcomes from this point of view. We argue that for PE to facilitate meaningful change in drug development, it is important to take the issue of democratization seriously and avoid attaching exclusively pragmatic significance to patients’ participation. Otherwise, in the absence of aspirations to democratization, PE in drug development risks devolving into a technical exercise, devoid of its hoped-for transformative powers.

## Limitations

Results of this review cannot be taken as a direct representation of the state of PE in drug development. We mapped PE in drug development based on the accounts published in academic journals between 2011 and 2021 in English. Conference abstracts were not included because details they provide about PE initiatives tend to be insufficient for the purposes of this review; also, the dispersed body of grey literature remained untouched. Thus, instances of PE described in the venues other than academic journals and in languages other than English are not included in this review, impacting the picture obtained. Further, it is conceivable that many instances of PE, especially the ones conducted by the corporate actors, remain unpublished and, therefore, not reflected in this review. Diverse terminology used in the recent scholarship on PE may have resulted in relevant initiatives escaping our attention. Finally, this review focused on engagement of adult patients. Therefore, important initiatives to engage pediatric populations in drug development and possible efforts to engage more general public are not included.

In view of these limitations, it is important to further study the landscape of PE in drug development in its entirety. Recently the broader scholarship on public participation has moved from studying individual cases of participation to considering more holistically how diverse forms of participation interrelate in wider systems. By joining this “systemic turn”, studies of PE in drug development would make the next step towards understanding multiple collectives and spaces of PE and their interactions with broader political landscapes.

## Supplementary Information

**Additional file 1.** Summary of search strategies.

**Additional file 2.** Flow Diagram of identified, screened and eligible publications.

**Additional file 3.** Data extraction spreadsheet.

**Additional file 4.** Full list of included articles.

## Data Availability

Original articles are available through their respective publishers, some as open access.

## Abbreviations

EMBASE: Excerpta Medica Database
EUPATI: European Patients’ Academy on Therapeutic Innovation
FDA: Food and Drug Administration
MeSH: Medical subject headings
PE: Patient engagement
PFDD: Patient-Focused Drug Development
PRISMA-P: Preferred Reporting Items for Systematic Reviews and Meta-Analyses Protocols
R&D: Research and development
