# Supplementary material for: Practices of patient engagement in drug development: a systematic scoping review
Source: Res Involv Engagem. 2022 Jun 29;8:29. doi: 10.1186/s40900-022-00364-8 (PMC9243835; doi:10.1186/s40900-022-00364-8)
Supplement: Supplementary file 1 — Additional file 1. Summary of search strategies. [file 40900_2022_364_MOESM1_ESM.docx]

**Practices of patient engagement in drug development: A systematic review**

**Supplementary material: search strategy**

1. **Search-Important parameters (MeSH Terms; Included in other search terms):**

| Study Population (P) | Interest/ Indication (I) | Outcome (O) | Context (C) |
| --- | --- | --- | --- |
| -patient (*)  -patient organi#ation*  -patient network*  -patient advocate*  -patient advocate organi*  -patient group*  -patient representative*  -individual (*)  -volunteer*  -user*  -citizen*  -participant (*)  -consumer (*)  -personal | -early research  -preclinical  -preclinical stud*  -preclinical stage  -preclinical phase*  -preclinical research | -involvement  -engagement  -participation  -centricity  -voice (*)  -collaboration (*)  -consultation (*)  -partnership*  -leadership  -empowerment  -governance -preference (*)  -expectation (*)  -view (*)  -priorit*  -interest*  -knowledge  -expertise  -thought (*)  -insight*  -information  -opinion (*)  -feedback  -aspiration (*)  -burden*  -need*  -value*  -experience* -input*  -patient-reported  outcome (*)  -patient-centered outcome* | -drug development  -patient-centered drug development  -patient-focused drug development  -drug research  -research of drug*  -medical research  -medicine development  -medication development  -medicine deliver*  -drug delivery  -drug discover*  -drug discover* project*  -treatment development  -treatment discover*  -translational medical research  -drug evaluation  -treatment evaluation |

1. **Separate databases search:**

**PubMed**

(("patient participation"[MeSH Terms]) AND ("drug development"[MeSH Terms])) OR (((((((((((((((((((((((((((((((((((((("patient participation"[All Fields]) OR ("patient advocacy"[All Fields])) OR ("patient involvement"[All Fields])) OR ("patient engagement"[All Fields])) OR ("patient centricity"[All Fields])) OR ("patient voice"[All Fields])) OR ("patient voices"[All Fields])) OR ("patient collaboration"[All Fields])) OR ("patient consultation"[All Fields])) OR ("patient partnership"[All Fields])) OR ("patient leadership"[All Fields])) OR ("patient empowerment"[All Fields])) OR ("patient governance"[All Fields]))) OR ("patients' preferences"[All Fields])) OR ("patients' expectations"[All Fields])) OR ("patients' views"[All Fields])) OR ("patients' priorities"[All Fields])) OR ("patients' interests"[All Fields])) OR ("patient knowledge"[All Fields])) OR ("patient expertise"[All Fields])) OR ("patients' thoughts"[All Fields])) OR ("patient insight"[All Fields])) OR ("patient information"[All Fields])) OR ("patients' opinions"[All Fields])) OR ("patient feedback"[All Fields])) OR ("patients' aspirations"[All Fields])) OR ("patients' burdens"[All Fields])) OR ("patients' needs"[All Fields])) OR ("patients' values"[All Fields])) OR ("patients' experiences"[All Fields])) OR ("patient experience data"[All Fields])) OR ("patients' input"[All Fields])) OR ("patient-reported outcomes"[All Fields])) OR ("patient-centered outcomes"[All Fields])))) AND (((((((((((((((("early drug research"[All Fields]) OR ("preclinical research"[All Fields])) OR ("early drug development"[All Fields])) OR ("preclinical drug research"[All Fields])) OR (preclinical))) OR ("clinical trials"[All Fields])) OR ("clinical studies"[All Fields])) OR ("phase 1"[All Fields])) OR ("phase 2"[All Fields])) OR ("phase 3"[All Fields])) OR ("lead identification"[All Fields])) OR ("lead optimization"[All Fields])) OR ("candidate validation"[All Fields])) OR ("target product profile"[All Fields])) OR (target value profile)))—**6,362 results**

- English=**6,164 results**
- Publication date (the last 10 years) =**4,357 results**
- Free full text= **2,597 results**
- Case Reports/ Clinical Conference/ Clinical Study/ Clinical Trial= **538 results**
- De-duplication= **305 results**

**EMBASE and Ovid MEDLINE(R)**

('patient participation'/mj" **OR** 'patient involvement'/mj" **OR** 'patient engagement'/mj" **OR** 'patient centricity'/mj" **OR** 'patient* organi?zation*'/mj" **OR** 'patient* voice*'/mj" **OR** 'patient collaboration'/mj" **OR** 'patient advocacy'/mj" **OR** 'patient consultation'/mj" **OR** 'patient partnership'/mj" **OR** 'patient leadership'/mj" **OR** 'patient empowerment'/mj" **OR** 'patient governance'/mj" **OR** 'patient* preference*'/mj" **OR** 'patient* expectation*'/mj" **OR** 'patient* view*'/mj" **OR** 'patient* priorit*'/mj" **OR** 'patient* interest*'/mj" **OR** 'patient knowledge'/mj" **OR** 'patient expertise'/mj" **OR** 'patient* thought*'/mj" **OR** 'patient* insight*'/mj" **OR** 'patient information'/mj" **OR** 'patient* opinion*'/mj" **OR** 'patient feedback'/mj" **OR** 'patient* aspiration*'/mj" **OR** 'patient* burden*'/mj" **OR** 'patient* need*'/mj" **OR** 'patient* value*'/mj" **OR** 'patient* experience*'/mj" **OR** 'patient* input*'/mj" **OR** 'patient-reported outcome*'/mj" **OR** 'patient-centered outcome*'/mj" **OR** 'patient* testimon*'/mj")

**AND** ('drug development'/mj" **OR** 'drug research'/mj" **OR** 'preclinical'/mj" **OR** 'drug evaluation'/mj" **OR** 'clinical trial*'/mj" **OR** 'clinical stud*'/mj" **OR** 'phase 1'/mj" **OR** 'phase 2'/mj" **OR** 'phase 3'/mj" **OR** 'lead identification'/mj" **OR** 'lead optimization'/mj" **OR** 'target value profile'/mj" **OR** 'target product profile'/mj" **OR** 'candidate validation'/mj") 🡪 **2552 results**

**🡪** NOT review* = **2025 results**

🡪 limit to English language = **1944 results**

**🡪** limit to year="2011 - 2021" = **1421 results**

🡪deduplication = **1263 results**

1 ..nlpx "query='patient participation'/mj","desiredResults=10000","minHitsDivisor=7","permitHyponyms=NO","lowestVocabularySearchLevel=none","phrasesBroken=NO","speedWanted=Fastest","comment=No Related Terms","elimEnable=NO","constraintMinTerms=2" 3453

2 ..nlpx "query='patient involvement'/mj","desiredResults=10000","minHitsDivisor=7","permitHyponyms=NO","lowestVocabularySearchLevel=none","phrasesBroken=NO","speedWanted=Fastest","comment=No Related Terms","elimEnable=NO","constraintMinTerms=2" 1788

3 ..nlpx "query='patient engagement'/mj","desiredResults=10000","minHitsDivisor=7","permitHyponyms=NO","lowestVocabularySearchLevel=none","phrasesBroken=NO","speedWanted=Fastest","comment=No Related Terms","elimEnable=NO","constraintMinTerms=2" 8343

4 ..nlpx "query='patient centricity'/mj","desiredResults=10000","minHitsDivisor=7","permitHyponyms=NO","lowestVocabularySearchLevel=none","phrasesBroken=NO","speedWanted=Fastest","comment=No Related Terms","elimEnable=NO","constraintMinTerms=2" 471

5 ..nlpx "query='patient* voice*'/mj","desiredResults=10000","minHitsDivisor=7","permitHyponyms=NO","lowestVocabularySearchLevel=none","phrasesBroken=NO","speedWanted=Fastest","comment=No Related Terms","elimEnable=NO","constraintMinTerms=2" 6942

6 ..nlpx "query='patient collaboration'/mj","desiredResults=10000","minHitsDivisor=7","permitHyponyms=NO","lowestVocabularySearchLevel=none","phrasesBroken=NO","speedWanted=Fastest","comment=No Related Terms","elimEnable=NO","constraintMinTerms=2" 5634

7 ..nlpx "query='patient consultation'/mj","desiredResults=10000","minHitsDivisor=7","permitHyponyms=NO","lowestVocabularySearchLevel=none","phrasesBroken=NO","speedWanted=Fastest","comment=No Related Terms","elimEnable=NO","constraintMinTerms=2" 10787

8 ..nlpx "query='patient partnership'/mj","desiredResults=10000","minHitsDivisor=7","permitHyponyms=NO","lowestVocabularySearchLevel=none","phrasesBroken=NO","speedWanted=Fastest","comment=No Related Terms","elimEnable=NO","constraintMinTerms=2" 4235

9 ..nlpx "query='patient leadership'/mj","desiredResults=10000","minHitsDivisor=7","permitHyponyms=NO","lowestVocabularySearchLevel=none","phrasesBroken=NO","speedWanted=Fastest","comment=No Related Terms","elimEnable=NO","constraintMinTerms=2" 1788

10 ..nlpx "query='patient empowerment'/mj","desiredResults=10000","minHitsDivisor=7","permitHyponyms=NO","lowestVocabularySearchLevel=none","phrasesBroken=NO","speedWanted=Fastest","comment=No Related Terms","elimEnable=NO","constraintMinTerms=2" 4814

11 ..nlpx "query='patient governance'/mj","desiredResults=10000","minHitsDivisor=7","permitHyponyms=NO","lowestVocabularySearchLevel=none","phrasesBroken=NO","speedWanted=Fastest","comment=No Related Terms","elimEnable=NO","constraintMinTerms=2" 2008

12 ..nlpx "query='patient* preference*'/mj","desiredResults=10000","minHitsDivisor=7","permitHyponyms=NO","lowestVocabularySearchLevel=none","phrasesBroken=NO","speedWanted=Fastest","comment=No Related Terms","elimEnable=NO","constraintMinTerms=2" 5264

13 ..nlpx "query='patient* expectation*'/mj","desiredResults=10000","minHitsDivisor=7","permitHyponyms=NO","lowestVocabularySearchLevel=none","phrasesBroken=NO","speedWanted=Fastest","comment=No Related Terms","elimEnable=NO","constraintMinTerms=2" 5094

14 ..nlpx "query='patient* view*'/mj","desiredResults=10000","minHitsDivisor=7","permitHyponyms=NO","lowestVocabularySearchLevel=none","phrasesBroken=NO","speedWanted=Fastest","comment=No Related Terms","elimEnable=NO","constraintMinTerms=2" 1788

15 ..nlpx "query='patient* priorit*'/mj","desiredResults=10000","minHitsDivisor=7","permitHyponyms=NO","lowestVocabularySearchLevel=none","phrasesBroken=NO","speedWanted=Fastest","comment=No Related Terms","elimEnable=NO","constraintMinTerms=2" 270

16 ..nlpx "query='patient* interest*'/mj","desiredResults=10000","minHitsDivisor=7","permitHyponyms=NO","lowestVocabularySearchLevel=none","phrasesBroken=NO","speedWanted=Fastest","comment=No Related Terms","elimEnable=NO","constraintMinTerms=2" 6046

17 ..nlpx "query='patient knowledge'/mj","desiredResults=10000","minHitsDivisor=7","permitHyponyms=NO","lowestVocabularySearchLevel=none","phrasesBroken=NO","speedWanted=Fastest","comment=No Related Terms","elimEnable=NO","constraintMinTerms=2" 13311

18 ..nlpx "query='patient expertise'/mj","desiredResults=10000","minHitsDivisor=7","permitHyponyms=NO","lowestVocabularySearchLevel=none","phrasesBroken=NO","speedWanted=Fastest","comment=No Related Terms","elimEnable=NO","constraintMinTerms=2" 3335

19 ..nlpx "query='patient* thought*'/mj","desiredResults=10000","minHitsDivisor=7","permitHyponyms=NO","lowestVocabularySearchLevel=none","phrasesBroken=NO","speedWanted=Fastest","comment=No Related Terms","elimEnable=NO","constraintMinTerms=2" 1788

20 ..nlpx "query='patient* insight*'/mj","desiredResults=10000","minHitsDivisor=7","permitHyponyms=NO","lowestVocabularySearchLevel=none","phrasesBroken=NO","speedWanted=Fastest","comment=No Related Terms","elimEnable=NO","constraintMinTerms=2" 3809

21 ..nlpx "query='patient information'/mj","desiredResults=10000","minHitsDivisor=7","permitHyponyms=NO","lowestVocabularySearchLevel=none","phrasesBroken=NO","speedWanted=Fastest","comment=No Related Terms","elimEnable=NO","constraintMinTerms=2" 1788

22 ..nlpx "query='patient* opinion*'/mj","desiredResults=10000","minHitsDivisor=7","permitHyponyms=NO","lowestVocabularySearchLevel=none","phrasesBroken=NO","speedWanted=Fastest","comment=No Related Terms","elimEnable=NO","constraintMinTerms=2" 4898

23 ..nlpx "query='patient feedback'/mj","desiredResults=10000","minHitsDivisor=7","permitHyponyms=NO","lowestVocabularySearchLevel=none","phrasesBroken=NO","speedWanted=Fastest","comment=No Related Terms","elimEnable=NO","constraintMinTerms=2" 6725

24 ..nlpx "query='patient* aspiration*'/mj","desiredResults=10000","minHitsDivisor=7","permitHyponyms=NO","lowestVocabularySearchLevel=none","phrasesBroken=NO","speedWanted=Fastest","comment=No Related Terms","elimEnable=NO","constraintMinTerms=2" 13355

25 ..nlpx "query='patient* burden*'/mj","desiredResults=10000","minHitsDivisor=7","permitHyponyms=NO","lowestVocabularySearchLevel=none","phrasesBroken=NO","speedWanted=Fastest","comment=No Related Terms","elimEnable=NO","constraintMinTerms=2" 1788

26 ..nlpx "query='patient* need*'/mj","desiredResults=10000","minHitsDivisor=7","permitHyponyms=NO","lowestVocabularySearchLevel=none","phrasesBroken=NO","speedWanted=Fastest","comment=No Related Terms","elimEnable=NO","constraintMinTerms=2" 1788

27 ..nlpx "query='patient* value*'/mj","desiredResults=10000","minHitsDivisor=7","permitHyponyms=NO","lowestVocabularySearchLevel=none","phrasesBroken=NO","speedWanted=Fastest","comment=No Related Terms","elimEnable=NO","constraintMinTerms=2" 9826

28 ..nlpx "query='patient* experience*'/mj","desiredResults=10000","minHitsDivisor=7","permitHyponyms=NO","lowestVocabularySearchLevel=none","phrasesBroken=NO","speedWanted=Fastest","comment=No Related Terms","elimEnable=NO","constraintMinTerms=2" 9594

29 ..nlpx "query='patient* input*'/mj","desiredResults=10000","minHitsDivisor=7","permitHyponyms=NO","lowestVocabularySearchLevel=none","phrasesBroken=NO","speedWanted=Fastest","comment=No Related Terms","elimEnable=NO","constraintMinTerms=2" 4626

30 ..nlpx "query='patient-reported outcome*'/mj","desiredResults=10000","minHitsDivisor=7","permitHyponyms=NO","lowestVocabularySearchLevel=none","phrasesBroken=NO","speedWanted=Fastest","comment=No Related Terms","elimEnable=NO","constraintMinTerms=2" 5490

31 ..nlpx "query='patient-centered outcome*'/mj","desiredResults=10000","minHitsDivisor=7","permitHyponyms=NO","lowestVocabularySearchLevel=none","phrasesBroken=NO","speedWanted=Fastest","comment=No Related Terms","elimEnable=NO","constraintMinTerms=2" 6108

32 ..nlpx "query='patient* testimon*'/mj","desiredResults=10000","minHitsDivisor=7","permitHyponyms=NO","lowestVocabularySearchLevel=none","phrasesBroken=NO","speedWanted=Fastest","comment=No Related Terms","elimEnable=NO","constraintMinTerms=2" 270

33 ..nlpx "query='drug development'/mj","desiredResults=10000","minHitsDivisor=7","permitHyponyms=NO","lowestVocabularySearchLevel=none","phrasesBroken=NO","speedWanted=Fastest","comment=No Related Terms","elimEnable=NO","constraintMinTerms=2" 5748

34 ..nlpx "query='drug research'/mj","desiredResults=10000","minHitsDivisor=7","permitHyponyms=NO","lowestVocabularySearchLevel=none","phrasesBroken=NO","speedWanted=Fastest","comment=No Related Terms","elimEnable=NO","constraintMinTerms=2" 15300

35 ..nlpx "query='medical research'/mj","desiredResults=10000","minHitsDivisor=7","permitHyponyms=NO","lowestVocabularySearchLevel=none","phrasesBroken=NO","speedWanted=Fastest","comment=No Related Terms","elimEnable=NO","constraintMinTerms=2" 3610

36 ..nlpx "query='preclinical'/mj","desiredResults=10000","minHitsDivisor=7","permitHyponyms=NO","lowestVocabularySearchLevel=none","phrasesBroken=NO","speedWanted=Fastest","comment=No Related Terms","elimEnable=NO","constraintMinTerms=2" 19984

37 ..nlpx "query='early drug research'/mj","desiredResults=10000","minHitsDivisor=7","permitHyponyms=NO","lowestVocabularySearchLevel=none","phrasesBroken=NO","speedWanted=Fastest","comment=No Related Terms","elimEnable=NO","constraintMinTerms=2" 16701

38 ..nlpx "query='drug evaluation'/mj","desiredResults=10000","minHitsDivisor=7","permitHyponyms=NO","lowestVocabularySearchLevel=none","phrasesBroken=NO","speedWanted=Fastest","comment=No Related Terms","elimEnable=NO","constraintMinTerms=2" 771

39 ..nlpx "query='clinical trial*'/mj","desiredResults=10000","minHitsDivisor=7","permitHyponyms=NO","lowestVocabularySearchLevel=none","phrasesBroken=NO","speedWanted=Fastest","comment=No Related Terms","elimEnable=NO","constraintMinTerms=2" 14823

40 ..nlpx "query='clinical stud*'/mj","desiredResults=10000","minHitsDivisor=7","permitHyponyms=NO","lowestVocabularySearchLevel=none","phrasesBroken=NO","speedWanted=Fastest","comment=No Related Terms","elimEnable=NO","constraintMinTerms=2" 3669

41 ..nlpx "query='patient* organi?zation*'/mj","desiredResults=10000","minHitsDivisor=7","permitHyponyms=NO","lowestVocabularySearchLevel=none","phrasesBroken=NO","speedWanted=Fastest","comment=No Related Terms","elimEnable=NO","constraintMinTerms=2" 7106

42 ..nlpx "query='phase 1'/mj","desiredResults=10000","minHitsDivisor=7","permitHyponyms=NO","lowestVocabularySearchLevel=none","phrasesBroken=NO","speedWanted=Fastest","comment=No Related Terms","elimEnable=NO","constraintMinTerms=2" 6289

43 ..nlpx "query='phase 2'/mj","desiredResults=10000","minHitsDivisor=7","permitHyponyms=NO","lowestVocabularySearchLevel=none","phrasesBroken=NO","speedWanted=Fastest","comment=No Related Terms","elimEnable=NO","constraintMinTerms=2" 8791

44 ..nlpx "query='phase 3'/mj","desiredResults=10000","minHitsDivisor=7","permitHyponyms=NO","lowestVocabularySearchLevel=none","phrasesBroken=NO","speedWanted=Fastest","comment=No Related Terms","elimEnable=NO","constraintMinTerms=2" 2661

45 ..nlpx "query='lead identification'/mj","desiredResults=10000","minHitsDivisor=7","permitHyponyms=NO","lowestVocabularySearchLevel=none","phrasesBroken=NO","speedWanted=Fastest","comment=No Related Terms","elimEnable=NO","constraintMinTerms=2" 34523

46 ..nlpx "query='lead optimization'/mj","desiredResults=10000","minHitsDivisor=7","permitHyponyms=NO","lowestVocabularySearchLevel=none","phrasesBroken=NO","speedWanted=Fastest","comment=No Related Terms","elimEnable=NO","constraintMinTerms=2" 3204

47 ..nlpx "query='target value profile'/mj","desiredResults=10000","minHitsDivisor=7","permitHyponyms=NO","lowestVocabularySearchLevel=none","phrasesBroken=NO","speedWanted=Fastest","comment=No Related Terms","elimEnable=NO","constraintMinTerms=2" 7075

48 ..nlpx "query='target product profile'/mj","desiredResults=10000","minHitsDivisor=7","permitHyponyms=NO","lowestVocabularySearchLevel=none","phrasesBroken=NO","speedWanted=Fastest","comment=No Related Terms","elimEnable=NO","constraintMinTerms=2" 2763

49 ..nlpx "query='candidate validation'/mj","desiredResults=10000","minHitsDivisor=7","permitHyponyms=NO","lowestVocabularySearchLevel=none","phrasesBroken=NO","speedWanted=Fastest","comment=No Related Terms","elimEnable=NO","constraintMinTerms=2" 2946

50 ..nlpx "query='patient advocacy'/mj","desiredResults=10000","minHitsDivisor=7","permitHyponyms=NO","lowestVocabularySearchLevel=none","phrasesBroken=NO","speedWanted=Fastest","comment=No Related Terms","elimEnable=NO","constraintMinTerms=2" 2349

51 1 or 2 or 3 or 4 or 5 or 6 or 7 or 8 or 9 or 10 or 11 or 12 or 13 or 14 or 15 or 16 or 17 or 18 or 19 or 20 or 21 or 22 or 23 or 24 or 25 or 26 or 27 or 28 or 29 or 30 or 31 or 32 or 41 or 50 131672

52 33 or 34 or 35 or 36 or 37 or 38 or 39 or 40 or 42 or 43 or 44 or 45 or 46 or 47 or 48 or 49 138816

53 51 and 52 **2552**

54 NOT review*  **2025**

55 limit 54 to english language **1944**

56 limit 55 to yr="2011 - 2021" **1421**

**Deduplication: 1204**

**Web of Science**

ALL=(((((((((((((((((((((((((((((((((patient involvement) OR (patient engagement)) OR (patient participation)) OR (patient advocacy)) OR (patient centricity)) OR (patient* voice*)) OR (patient collaboration)) OR (patient consultation)) OR (patient partnership)) OR (patient leadership)) OR (patient empowerment)) OR (patient governance)) OR (patient* preference*)) OR (patient* expectation*)) OR (patient* view*)) OR (patient* priorit*)) OR (patient* interest*)) OR (patient knowledge)) OR (patient* expertise)) OR (patient* thought*)) OR (patient* insight*)) OR (patient* information)) OR (patient* opinion*)) OR (patient* feedback)) OR (patient* aspiration*)) OR (patient* burden*)) OR (patient* need*)) OR (patient* value*)) OR (patient* experience*)) OR (patient* input*)) OR (patient-reported outcome*)) OR (patient-centered outcome*)) OR (patient* testimon*)) 🡪 **all fields**

AND

AK=(((((((((((((((drug research) OR (drug development)) OR (pharmaceutical research)) OR (pharmaceutical development)) OR (preclinical)) OR (clinical trial*)) OR (clinical stud*)) OR (phase 1)) OR (phase 2)) OR (phase 3)) OR (lead identification)) OR (lead optimization)) OR (candidate validation)) OR (target value profile)) OR (target product profile))🡪 **keywords specified by authors of the articles**

TOTAL: **19,907 results**

Language (English): **19,297 results**

Timespan (2011-2021): **13,337 results**

NOT (narrative review* OR systematic review*): **12,605 results**

Document type (article OR meeting abstract OR proceedings paper OR early access OR editorial material): **10,198 results**

Open access: **5,490 results**

Source titles (trials OR clinical therapeutics OR contemporary clinical trials OR contemporary clinical trials communications OR clinical trials OR investigational new drugs OR JMIR research protocols OR Journal of Translational medicine OR cancer chemotherapy and pharmacology OR frontiers in pharmacology OR British Journal of Clinical Pharmacology): **552 results**
