# Supplementary material for: Practices of patient engagement in drug development: a systematic scoping review
Source: Res Involv Engagem. 2022 Jun 29;8:29. doi: 10.1186/s40900-022-00364-8 (PMC9243835; doi:10.1186/s40900-022-00364-8)
Supplement: Supplementary file 2 — Additional file 2. Flow Diagram of identified, screened and eligible publications. [file 40900_2022_364_MOESM2_ESM.docx]

Appendix 1. PRISMA flow diagram.

**Identification of studies via databases**

**Identification of studies via another method**

Reports excluded:

(n= 117)

Non-empirical articles (n = 62)
No Drug Development (n = 22)

No Patient Engagement (n = 20)

Abstract only (n = 11)

Pediatrics (n = 2)

Records removed *before screening*:

Not in English (n = 889)

Not published between 2011-2021 (n = 8,290)

Not in full text (n = 6,468)

Unsuitable document type (10,653)
Duplicate records (n = 460)

Records identified from the citation search (n = 138)

Records identified from:

EMBASE (n = 2,552)

Web of Science (n = 19,907)

PubMed (n = 6,362)

Total (n = 28,821)

**Identification**

Records excluded in the 1^st^ round of title and abstract screening (n = 1,396)

Records screened (n = 2,061)

Reports assessed for eligibility

(n = 665)

Reports excluded:

In the 2^nd^ round (n = 297)

**Screening**

Included studies from databases (n = 368)
Included studies from the citation search (n = 21)

Reports excluded:

In the 3^rd^ round (n = 294)
In the 4^th^ round (n = 15)

In the 5^th^ round (n = 11)

Total studies included in the review (n = 69)

**Included**

Appendix 1. PRISMA flow diagram.

**Identification of studies via databases**

**Identification of studies via another method**

Reports excluded:

(n= 117)

Non-empirical articles (n = 62)
No Drug Development (n = 22)

No Patient Engagement (n = 20)

Abstract only (n = 11)

Pediatrics (n = 2)

Records excluded in the 1^st^ round of title and abstract screening (n = 1,396)

Records identified from the citation search (n = 138)

Records identified from:

EMBASE (n = 1,204)

Web of Science (n = 552)

PubMed (n = 305)

Total (n = 2,601)

**Identification**

Reports excluded:

In the 2^nd^ round (n = 297)

Reports assessed for eligibility

(n = 665)

**Screening**

Included studies from databases (n = 368)
Included studies from the citation search (n = 21)

Reports excluded:

In the 3^rd^ round (n = 294)
In the 4^th^ round (n = 15)

In the 5^th^ round (n = 11)

Total studies included in the review (n = 69)

**Included**

| 1^st^ round of exclusion | -Abstract only, poster or conference presentations;  -Biomedical in-vitro/laboratory studies;  -Trials on medical devices, drug delivery systems, nutritional supplements, drug prescription practices or already approved drugs;  -Trial reports/study protocols without explicit patient engagement;  -Biomarker studies;  -Insignificant patient engagement activities, such as reporting adverse events (that were not always specified as patient-reported);  -Studies focused on healthcare;  -Pragmatic trials;  -Non-empirical articles;  -Pediatric trials;  -Policy prescriptions |
| --- | --- |
| 2^nd^ round of exclusion | -Clinical trials that address PROs as secondary or exploratory outcomes;  -Articles that engage predominantly doctors, providers or site investigators and not patients |
| 3^rd^ round of exclusion | -Articles that are extensions of a different study and do not have a unique study protocol/secondary analysis; |
| 4^th^ round of exclusion | -Community-based articles;  -Articles that use ACR20 as PROs. ACR20 is considered a traditional clinical endpoint and not exactly a PRO, although it seemingly contains several PROs. (American College of Rheumatology 20 criteria that looks at how many patients with Rheumatoid Arthritis achieve a 20% improvement in tender or swollen joint counts, as well as other patient-related criteria: patient assessment, pain scale and disability/functional questionnaire. |
| 5^th^ round of exclusion | -Weak intention of changing practice, without providing concrete recommendations for improving patient engagement |
