# Supplementary material for: Practices of patient engagement in drug development: a systematic scoping review
Source: Res Involv Engagem. 2022 Jun 29;8:29. doi: 10.1186/s40900-022-00364-8 (PMC9243835; doi:10.1186/s40900-022-00364-8)
Supplement: Supplementary file 4 — Additional file 4. Full list of included articles. [file 40900_2022_364_MOESM4_ESM.docx]

***Finetuning details-Consulting***

1. Breitkopf, C. R., Ridgeway, J. L., Asiedu, G. B., Carroll, K., Tenney, M., & Jatoi, A. (2016). Ovarian cancer patients’ and their family members’ perspectives on novel vaccine and virotherapy trials. In *CLINICAL TRIALS* (Vol. 13, Issue 6, pp. 660–664). SAGE PUBLICATIONS LTD. <https://doi.org/10.1177/1740774516654445>
2. Bygrave C., Burton T., Carter P., Richard S., Low E., & Bloodworth C. (2017). Patient perception of being involved in a clinical trial for multiple myeloma. *British Journal of Haematology*, *176*(Supplement 1), 80. <https://doi.org/10.1111/bjh.14613>
3. Dias, A. L., Chao, J. H., Lee, D., Wu, Y., & Kloecker, G. H. (2016). Patient perceptions concerning clinical trials in oncology patients. In *CONTEMPORARY CLINICAL TRIALS COMMUNICATIONS* (Vol. 4, pp. 179–185). ELSEVIER INC. <https://doi.org/10.1016/j.conctc.2016.09.005>
4. Geana, M., Erba, J., Krebill, H., Doolittle, G., Madhusudhana, S., Qasem, A., Malomo, N., & Sharp, D. (2017). Searching for cures: Inner-city and rural patients’ awareness and perceptions of cancer clinical trials. In *CONTEMPORARY CLINICAL TRIALS COMMUNICATIONS* (Vol. 5, pp. 72–79). ELSEVIER INC. <https://doi.org/10.1016/j.conctc.2016.12.004>
5. Hillyer, G. C., Beauchemin, M., Hershman, D. L., Kelsen, M., Brogan, F. L., Sandoval, R., Schmitt, K. M., Reyes, A., Terry, M. B., Lassman, A. B., & Schwartz, G. K. (2020). Discordant attitudes and beliefs about cancer clinical trial participation between physicians, research staff, and cancer patients. In *CLINICAL TRIALS* (Vol. 17, Issue 2, pp. 184–194). SAGE PUBLICATIONS LTD. <https://doi.org/10.1177/1740774520901514>
6. Politi, M. C., Kuzemchak, M. D., Kaphingst, K. A., Perkins, H., Liu, J., & Byrne, M. M. (2016). Decision Aids Can Support Cancer Clinical Trials Decisions: Results of a Randomized Trial. *The Oncologist*, *21*(12), 1461–1470. <https://doi.org/10.1634/theoncologist.2016-0068>
7. Solic I., Stipcic A., Pavlicevic I., & Marusic A. (2017). Transparency and public accessibility of clinical trial information in Croatia: How it affects patient participation in clinical trials. *Biochemia Medica*, *27*(2), 259–269. <https://doi.org/10.11613/BM.2017.027>
8. Sygna, K., Johansen, S., & Ruland, C. M. (2015). Recruitment challenges in clinical research including cancer patients and their caregivers. A randomized controlled trial study and lessons learned. In *TRIALS* (Vol. 16). BMC. <https://doi.org/10.1186/s13063-015-0948-y>

***Finetuning details-Involving***

1. Brown, R. F., Davis, R., Wilson Genderson, M., Grant, S., Cadet, D., Lessard, M., Alpert, J., Ward, J., & Ginder, G. (2016). African-American patients with cancer Talking About Clinical Trials (TACT) with oncologists during consultations: Evaluating the efficacy of tailored health messages in a randomised controlled trial-the TACT study protocol. *BMJ Open*, *6*(12), e012864. <https://doi.org/10.1136/bmjopen-2016-012864>
2. Carney, P. A., Tucker, E. K., Newby, T. A., & Beer, T. M. (2014). Feasibility, acceptability and findings from a pilot randomized controlled intervention study on the impact of a book designed to inform patients about cancer clinical trials. *Journal of Cancer Education: The Official Journal of the American Association for Cancer Education*, *29*(1), 181–187. <https://doi.org/10.1007/s13187-013-0567-9>
3. Cartmell, K. B., Bonilha, H. S., Matson, T., Bryant, D. C., Zapka, J., Bentz, T. A., Ford, M. E., Hughes-Halbert, C., Simpson, K. N., & Alberg, A. J. (2016). Patient participation in cancer clinical trials: A pilot test of lay navigation. In *CONTEMPORARY CLINICAL TRIALS COMMUNICATIONS* (Vol. 3, pp. 86–93). ELSEVIER INC. <https://doi.org/10.1016/j.conctc.2016.04.005>
4. Dellson, P., Nilsson, K., Jernstrom, H., & Carlsson, C. (2018). Patients’ reasoning regarding the decision to participate in clinical cancer trials: An interview study. In *TRIALS* (Vol. 19). BMC. <https://doi.org/10.1186/s13063-018-2916-9>
5. Godskesen, T. M., Kihlbom, U., Nordin, K., Silen, M., & Nygren, P. (2016). Differences in trial knowledge and motives for participation among cancer patients in phase 3 clinical trials. *European Journal of Cancer Care*, *25*(3), 516–523. <https://doi.org/10.1111/ecc.12319>
6. Knapp, P., Raynor, D. K., Silcock, J., & Parkinson, B. (2011). Can user testing of a clinical trial patient information sheet make it fit-for-purpose? —A randomized controlled trial. *BMC Medicine*, *9*, 89. <https://doi.org/10.1186/1741-7015-9-89>
7. Meyers, K., Rodriguez, K., Brill, A. L., Wu, Y., La Mar, M., Dunbar, D., Koblin, B., Margolis, D., Sobieszczyk, M. E., Van Tieu, H., Frank, I., Markowitz, M., & Golub, S. A. (2018). Lessons for Patient Education Around Long-Acting Injectable PrEP: Findings from a Mixed-Method Study of Phase II Trial Participants. *AIDS and Behavior*, *22*(4), 1209–1216. <https://doi.org/10.1007/s10461-017-1871-x>
8. Uveges, M. K., Lansey, D. G., Mbah, O., Gray, T., Sherden, L., & Wenzel, J. (2018). Patient navigation and clinical trial participation: A randomized controlled trial design. In *CONTEMPORARY CLINICAL TRIALS COMMUNICATIONS* (Vol. 12, pp. 98–102). ELSEVIER INC. <https://doi.org/10.1016/j.conctc.2018.09.003>

***Designing studies-Consulting***

1. Basch, E., Autio, K., Ryan, C. J., Mulders, P., Shore, N., Kheoh, T., Fizazi, K., Logothetis, C. J., Rathkopf, D., Smith, M. R., Mainwaring, P. N., Hao, Y., Griffin, T., Li, S., Meyers, M. L., Molina, A., & Cleeland, C. (2013). Abiraterone acetate plus prednisone versus prednisone alone in chemotherapy-naive men with metastatic castration-resistant prostate cancer: Patient-reported outcome results of a randomised phase 3 trial. *The Lancet. Oncology*, *14*(12), 1193–1199. <https://doi.org/10.1016/S1470-2045(13)70424-8>
2. Dellon, E. S., Katzka, D. A., Collins, M. H., Hamdani, M., Gupta, S. K., & Hirano, I. (2017). Budesonide Oral Suspension Improves Symptomatic, Endoscopic, and Histologic Parameters Compared With Placebo in Patients With Eosinophilic Esophagitis. *Gastroenterology*, *152*(4), 776-786.e5. <https://doi.org/10.1053/j.gastro.2016.11.021>
3. Dueck, A. C., Scher, H. I., Bennett, A. V., Mazza, G. L., Thanarajasingam, G., Schwab, G., Weitzman, A. L., Rogak, L. J., & Basch, E. (2020). Assessment of Adverse Events From the Patient Perspective in a Phase 3 Metastatic Castration-Resistant Prostate Cancer Clinical Trial. *JAMA Oncology*, *6*(2), e193332. <https://doi.org/10.1001/jamaoncol.2019.3332>
4. Jost W.H., Pagan F., Michel O., Oehlwein C., Slawek J., Bogucki A., Ochudlo S., Banach M., Flatau-Baque B., Csikos J., & Blitzer A. (2020). Long-term incobotulinumtoxinA treatment for chronic sialorrhea: Efficacy and safety over 64 weeks. *Parkinsonism and Related Disorders*, *70*, 23–30.
5. Kee Y.K., Han S.Y., Kang D.-H., Noh J.W., Jeong K.H., Kim G.-H., Kim Y.W., & Kim B.S. (2021). Comparison of different types of oral adsorbent therapy in patients with chronic kidney disease: A multicenter, randomized, phase iv clinical trial. *Yonsei Medical Journal*, *62*(1), 41–49. <https://doi.org/10.3349/ymj.2021.62.1.41>
6. Kennedy, F., Shearsmith, L., Ayres, M., Lindner, O. C., Marston, L., Pass, A., Danson, S., & Velikova, G. (n.d.). Online monitoring of patient self-reported adverse events in early phase clinical trials: Views from patients, clinicians, and trial staff. In *CLINICAL TRIALS*. SAGE PUBLICATIONS LTD. <https://doi.org/10.1177/1740774520972125>
7. Lattermann, C., Jacobs, C. A., Bunnell, M. P., Jochimsen, K. N., Abt, J. P., Reinke, E. K., Gammon, L. G., Huebner, J. L., Kraus, V. B., & Spindler, K. P. (2017). Logistical challenges and design considerations for studies using acute anterior cruciate ligament injury as a potential model for early posttraumatic osteoarthritis. *Journal of Orthopaedic Research: Official Publication of the Orthopaedic Research Society*, *35*(3), 641–650. <https://doi.org/10.1002/jor.23329>
8. Nipp, R. D., Lee, H., Powell, E., Birrer, N. E., Poles, E., Finkelstein, D., Winkfield, K., Percac-Lima, S., Chabner, B., & Moy, B. (2016). Financial Burden of Cancer Clinical Trial Participation and the Impact of a Cancer Care Equity Program. *The Oncologist*, *21*(4), 467–474. <https://doi.org/10.1634/theoncologist.2015-0481>
9. Sieper, J., Kivitz, A., van Tubergen, A., Deodhar, A., Coteur, G., Woltering, F., & Landewé, R. (2015). Impact of Certolizumab Pegol on Patient-Reported Outcomes in Patients With Axial Spondyloarthritis. *Arthritis Care & Research*, *67*(10), 1475–1480. <https://doi.org/10.1002/acr.22594>
10. Sticherling, M., Eicke, C., & Anger, T. (n.d.). *Practicability of combined treatment with calcipotriol/betamethasone gel (Daivobet® Gel) and improvement of quality of life in patients with psoriasis*.
11. Strand, V., Kremer, J., Wallenstein, G., Kanik, K. S., Connell, C., Gruben, D., Zwillich, S. H., & Fleischmann, R. (2015). Effects of tofacitinib monotherapy on patient-reported outcomes in a randomized phase 3 study of patients with active rheumatoid arthritis and inadequate responses to DMARDs. *Arthritis Research & Therapy*, *17*(101154438), 307. <https://doi.org/10.1186/s13075-015-0825-9>
12. Strand, V., van der Heijde, D., Tanaka, Y., Keystone, E., Kremer, J., Zerbini, C. A. F., Cardiel, M. H., Cohen, S., Nash, P., Song, Y.-W., Tegzova, D., Gruben, D., Wallenstein, G., Connell, C. A., Fleischmann, R., & ORAL Scan investigators. (2020). Tofacitinib in combination with methotrexate in patients with rheumatoid arthritis: Patient-reported outcomes from the 24-month Phase 3 ORAL Scan study. *Clinical and Experimental Rheumatology*, *38*(5), 848–857.
13. Strand, V., van Vollenhoven, R. F., Lee, E. B., Fleischmann, R., Zwillich, S. H., Gruben, D., Koncz, T., Wilkinson, B., & Wallenstein, G. (2016). Tofacitinib or adalimumab versus placebo: Patient-reported outcomes from a phase 3 study of active rheumatoid arthritis. *Rheumatology (Oxford, England)*, *55*(6), 1031–1041. <https://doi.org/10.1093/rheumatology/kev442>
14. Tabberer, M., Lomas, D. A., Birk, R., Brealey, N., Zhu, C.-Q., Pascoe, S., Locantore, N., & Lipson, D. A. (2018). Once-Daily Triple Therapy in Patients with COPD: Patient-Reported Symptoms and Quality of Life. *Advances in Therapy*, *35*(1), 56–71. <https://doi.org/10.1007/s12325-017-0650-4>
15. The Chronic GVHD Consortium. (2018). Design and Patient Characteristics of the Chronic Graft-versus-Host Disease Response Measures Validation Study. *Biology of Blood and Marrow Transplantation: Journal of the American Society for Blood and Marrow Transplantation*, *24*(8), 1727–1732. <https://doi.org/10.1016/j.bbmt.2018.02.010>
16. Burkhalter, J. E., Aboulafia, D. M., Botello-Harbaum, M., & Lee, J. Y. (2018). Participant characteristics and clinical trial decision-making factors in AIDS malignancy consortium treatment trials for HIV-infected persons with cancer (AMC #S006). *HIV Clinical Trials*, *19*(6), 235–241. <https://doi.org/10.1080/15284336.2018.1537349>

***Designing studies-Involving***

1. Allen, J., Child, A., & Mertens, S. (2019). The development of an end-to-end service solution to support lupus patients and improve their experience in clinical trials. In *CLINICAL TRIALS* (Vol. 16, Issue 1, pp. 71–80). SAGE PUBLICATIONS LTD. <https://doi.org/10.1177/1740774518811111>
2. Anderson A., Borfitz D., & Getz K. (2018). Global Public Attitudes About Clinical Research and Patient Experiences With Clinical Trials. *JAMA Network Open*, *1*(6), e182969. <https://doi.org/10.1001/jamanetworkopen.2018.2969>
3. Argov, Z., Caraco, Y., Lau, H., Pestronk, A., Shieh, P. B., Skrinar, A., Koutsoukos, T., Ahmed, R., Martinisi, J., & Kakkis, E. (2016). Aceneuramic Acid Extended Release Administration Maintains Upper Limb Muscle Strength in a 48-week Study of Subjects with GNE Myopathy: Results from a Phase 2, Randomized, Controlled Study. *Journal of Neuromuscular Diseases*, *3*(1), 49–66. <https://doi.org/10.3233/JND-159900>
4. Bonner, N., Abetz-Webb, L., Renault, L., Caballero, T., Longhurst, H., Maurer, M., Christiansen, S., & Zuraw, B. (2015). Development and content validity testing of a patient-reported outcomes questionnaire for the assessment of hereditary angioedema in observational studies. *Health and Quality of Life Outcomes*, *13*, 92. <https://doi.org/10.1186/s12955-015-0292-7>
5. Chiarotto, A., Terwee, C. B., Deyo, R. A., Boers, M., Lin, C.-W. C., Buchbinder, R., Corbin, T. P., Costa, L. O. P., Foster, N. E., Grotle, M., Koes, B. W., Kovacs, F. M., Maher, C. G., Pearson, A. M., Peul, W. C., Schoene, M. L., Turk, D. C., van Tulder, M. W., & Ostelo, R. W. (2014). A core outcome set for clinical trials on non-specific low back pain: Study protocol for the development of a core domain set. In *TRIALS* (Vol. 15). BMC. <https://doi.org/10.1186/1745-6215-15-511>
6. Chin, K. M., Gomberg-Maitland, M., Channick, R. N., Cuttica, M. J., Fischer, A., Frantz, R. P., Hunsche, E., Kleinman, L., McConnell, J. W., McLaughlin, V. V., Miller, C. E., Zamanian, R. T., Zastrow, M. S., & Badesch, D. B. (2018). Psychometric Validation of the Pulmonary Arterial Hypertension-Symptoms and Impact (PAH-SYMPACT) Questionnaire: Results of the SYMPHONY Trial. *Chest*, *154*(4), 848–861. <https://doi.org/10.1016/j.chest.2018.04.027>
7. Chiodini, P., Arenare, L., Piccirillo, M. C., Perrone, F., & Gallo, C. (2020). A phase 2, open label, multicenter, single arm study of tocilizumab on the efficacy and tolerability of tocilizumab in the treatment of patients with COVID-19 pneumonia (TOCIVID-19 trial): Statistical analysis plan. *Contemporary Clinical Trials Communications*, *20*, 100665. <https://doi.org/10.1016/j.conctc.2020.100665>
8. DesRuisseaux, L. A., Williams, V. J., McManus, A. J., Gupta, A. S., Carlyle, B. C., Azami, H., Gerber, J. A., Bolling, A. M., Cook, C. L., Betensky, R. A., & Arnold, S. E. (2020). A pilot protocol to assess the feasibility of a virtual multiple crossover, randomized controlled trial design using methylphenidate in mild cognitive impairment. In *TRIALS* (Vol. 21, Issue 1). BMC. <https://doi.org/10.1186/s13063-020-04752-x>
9. Eggly, S., Hamel, L. M., Heath, E., Manning, M. A., Albrecht, T. L., Barton, E., Wojda, M., Foster, T., Carducci, M., Lansey, D., Wang, T., Abdallah, R., Abrahamian, N., Kim, S., Senft, N., & Penner, L. A. (2017). Partnering around cancer clinical trials (PACCT): Study protocol for a randomized trial of a patient and physician communication intervention to increase minority accrual to prostate cancer clinical trials. *BMC Cancer*, *17*(1), 807. <https://doi.org/10.1186/s12885-017-3804-5>
10. Gordon M.F., Lenderking W.R., Duhig A., Chandler J., Lundy J.J., Miller D.S., Piault-Louis E., Doody R.S., Galasko D., Gauthier S., & Frank L. (2016). Development of a patient-reported outcome instrument to assess complex activities of daily living and interpersonal functioning in persons with mild cognitive impairment: The qualitative research phase. *Alzheimer’s and Dementia*, *12*(1), 75–84. <https://doi.org/10.1016/j.jalz.2015.04.008>
11. Henderson, G. E., Waltz, M., Meagher, K., Cadigan, R. J., Jupimai, T., Isaacson, S., Ormsby, N. Q., Colby, D. J., Kroon, E., Phanuphak, N., Ananworanich, J., & Peay, H. L. (2019). Going off antiretroviral treatment in a closely monitored HIV “cure” trial: Longitudinal assessments of acutely diagnosed trial participants and decliners. *Journal of the International AIDS Society*, *22*(3), e25260. <https://doi.org/10.1002/jia2.25260>
12. Hu, X., Zhang, C., & Zhang, Y. (2017). BC-PROM: validation of a patient-reported outcomes measure for patients with breast cancer. *Medicine*, *96*(17), e6781. <https://doi.org/10.1097/MD.0000000000006781>
13. Klopstock, T., Escolar, M. L., Marshall, R. D., Perez-Dueñas, B., Tuller, S., Videnovic, A., & Greblikas, F. (2019). The FOsmetpantotenate Replacement Therapy (FORT) randomized, double-blind, Placebo-controlled pivotal trial: Study design and development methodology of a novel primary efficacy outcome in patients with pantothenate kinase-associated neurodegeneration. *Clinical Trials (London, England)*, *16*(4), 410–418. <https://doi.org/10.1177/1740774519845673>
14. Koller, M., Hjermstad, M. J., Tomaszewski, K. A., Tomaszewska, I. M., Hornslien, K., Harle, A., Arraras, J. I., Morag, O., Pompili, C., Ioannidis, G., Georgiou, M., Navarra, C., Chie, W.-C., Johnson, C. D., Himpel, A., Schulz, C., Bohrer, T., Janssens, A., Kuliś, D., & Bottomley, A. (2017). An international study to revise the EORTC questionnaire for assessing quality of life in lung cancer patients. *Annals of Oncology: Official Journal of the European Society for Medical Oncology*, *28*(11), 2874–2881. <https://doi.org/10.1093/annonc/mdx453>
15. Racine, E., Hurley, C., Cheung, A., Sinnott, C., Matvienko-Sikar, K., Smithson, W. H., & Kearney, P. M. (2017). Study within a trial (SWAT) protocol. Participants’ perspectives and preferences on clinical trial result dissemination: The TRUST Thyroid Trial experience. In *CONTEMPORARY CLINICAL TRIALS COMMUNICATIONS* (Vol. 7, pp. 163–165). ELSEVIER INC. <https://doi.org/10.1016/j.conctc.2017.07.001>
16. Tattersall, M. H. N., Jefford, M., Martin, A., Olver, I., Thompson, J. F., Brown, R. F., & Butow, P. N. (2017). Parallel multicentre randomised trial of a clinical trial question prompt list in patients considering participation in phase 3 cancer treatment trials. *BMJ Open*, *7*(3), e012666. <https://doi.org/10.1136/bmjopen-2016-012666>
17. Theodore-Oklota C., Arbuckle R., Bonner N., & Spencer H. (2017). Qualitative research to explore the patient experience of x-linked hypophosphataemia (XLH) and to evaluate the content validity of the BPI-SF and womac for use as clinical trial endpoints. *Value in Health*, *20*(5), A331.

***Designing studies-Partnering***

1. Calis, K. A., Archdeacon, P., Bain, R. P., Forrest, A., Perlmutter, J., & DeMets, D. L. (2017). Understanding the functions and operations of data monitoring committees: Survey and focus group findings. In *CLINICAL TRIALS* (Vol. 14, Issue 1, pp. 59–66). SAGE PUBLICATIONS LTD. <https://doi.org/10.1177/1740774516679665>
2. Gillies, K., Skea, Z. C., Maclennan, S. J., Ramsay, C. R., & Campbell, M. K. (n.d.). *Determining information for inclusion in a decision-support intervention for clinical trial participation: A modified Delphi approach*.
3. Huang, G. D., Bull, J., McKee, K. J., Mahon, E., Harper, B., Roberts, J. N., & Team, C. R. P. (2018). Clinical trials recruitment planning: A proposed framework from the Clinical Trials Transformation Initiative. In *CONTEMPORARY CLINICAL TRIALS* (Vol. 66, pp. 74–79). ELSEVIER SCIENCE INC. <https://doi.org/10.1016/j.cct.2018.01.003>
4. Hutchison, C., Kwong, A., Ray, S., Struble, K., Swan, T., & Miller, V. (2014). Accelerating drug development through collaboration: The Hepatitis C Drug Development Advisory Group. *Clinical Pharmacology and Therapeutics*, *96*(2), 162–165. <https://doi.org/10.1038/clpt.2014.113>
5. LoRusso, S., Johnson, N. E., McDermott, M. P., Eichinger, K., Butterfield, R. J., Carraro, E., Higgs, K., Lewis, L., Mul, K., Sacconi, S., Sansone, V. A., Shieh, P., van Engelen, B., Wagner, K., Wang, L., Statland, J. M., & Tawil, R. (2019). Clinical trial readiness to solve barriers to drug development in FSHD (ReSolve): Protocol of a large, international, multi-center prospective study. *BMC Neurology*, *19*(1), 224. <https://doi.org/10.1186/s12883-019-1452-x>
6. Perry, B., Geoghegan, C., Lin, L., McGuire, H. F., Nido, V., Grabert, B., Morin, S. L., Hallinan, Z. P., & Corneli, A. (2019). Patient preferences for using mobile technologies in clinical trials. In *CONTEMPORARY CLINICAL TRIALS COMMUNICATIONS* (Vol. 15). ELSEVIER INC.
7. van Overbeeke E., Janssens R., Whichello C., Scholin Bywall K., Sharpe J., Nikolenko N., Phillips B.S., Guiddi P., Mazzocco K., Vergani L., Marton G., Cleemput I., Simoens S., Juhaeri J., Levitan B., Kubler J., de Bekker-Grob E.W., Veldwijk J., & Huys I. (2019). Design, Conduct, and Use of Patient Preference Studies in the Medical Product Life Cycle: A Multi-Method Study. *Value in Health*, *21*(Supplement 3), S390. <https://doi.org/10.1016/j.jval.2018.09.2317>

***Setting up research and development programs-Consulting***

1. Farmer, A., Aymé, S., de Heredia, M. L., Maffei, P., McCafferty, S., Młynarski, W., Nunes, V., Parkinson, K., Paquis-Flucklinger, V., Rohayem, J., Sinnott, R., Tillmann, V., Tranebjaerg, L., & Barrett, T. G. (2013). EURO-WABB: an EU rare diseases registry for Wolfram syndrome, Alström syndrome and Bardet-Biedl syndrome. *BMC Pediatrics*, *13*, 130. <https://doi.org/10.1186/1471-2431-13-130>
2. Patalano F., Gutzwiller F.S., Shah B., Kumari C., & Cook N.S. (2020). Gathering Structured Patient Insight to Drive the PRO Strategy in COPD: Patient-Centric Drug Development from Theory to Practice. *Advances in Therapy*, *37*(1), 17–26. <https://doi.org/10.1007/s12325-019-01134-x>

***Setting up research and development programs-Involving***

1. Blackwell T.S., Tager A.M., Borok Z., Moore B.B., Schwartz D.A., Anstrom K.J., Bar-Joseph Z., Bitterman P., Blackburn M.R., Bradford W., Brown K.K., Chapman H.A., Collard H.R., Cosgrove G.P., Deterding R., Doyle R., Flaherty K.R., Garcia C.K., Hagood J.S., … Eu J.P. (2014). Future directions in idiopathic pulmonary fibrosis research an NHLBI workshop report. *American Journal of Respiratory and Critical Care Medicine*, *189*(2), 214–222. <https://doi.org/10.1164/rccm.201306-1141WS>
2. Cook, N. S., Cave, J., & Holtorf, A.-P. (2019). Patient Preference Studies During Early Drug Development: Aligning Stakeholders to Ensure Development Plans Meet Patient Needs. *Frontiers in Medicine*, *6*(101648047), 82. <https://doi.org/10.3389/fmed.2019.00082>
3. Holmes, E. A. F., Plumpton, C., Baker, G. A., Jacoby, A., Ring, A., Williamson, P., Marson, A., & Hughes, D. A. (2019). Patient-Focused Drug Development Methods for Benefit-Risk Assessments: A Case Study Using a Discrete Choice Experiment for Antiepileptic Drugs. *Clinical Pharmacology and Therapeutics*, *105*(3), 672–683. <https://doi.org/10.1002/cpt.1231>
4. Manzi S. et al. (2019). Global consensus building and prioritisation of fundamental lupus challenges: The ALPHA project. *Arthritis and Rheumatology*, *6*(1). <https://lupus.bmj.com/content/6/1/e000342>
5. Postmus D., Mavris M., Hillege H.L., Salmonson T., Ryll B., Plate A., Moulon I., Eichler H.-G., Bere N., & Pignatti F. (2016). Incorporating patient preferences into drug development and regulatory decision making: Results from a quantitative pilot study with cancer patients, carers, and regulators. *Clinical Pharmacology and Therapeutics*, *99*(5), 548–554. <https://doi.org/10.1002/cpt.332>

***Setting up research and development programs-Partnering***

1. Evon, D. M., Golin, C. E., Stewart, P., Fried, M. W., Alston, S., Reeve, B., Lok, A. S., Sterling, R. K., Lim, J. K., Reau, N., Sarkar, S., Nelson, D. R., Reddy, K. R., & Di Bisceglie, A. M. (2017). Patient engagement and study design of PROP UP: A multi-site patient-centered prospective observational study of patients undergoing hepatitis C treatment. *Contemporary Clinical Trials*, *57*, 58–68. <https://doi.org/10.1016/j.cct.2017.03.013>
2. Lovell, N., Etkind, S. N., Bajwah, S., Maddocks, M., & Higginson, I. J. (2020). What influenced people with chronic or refractory breathlessness and advanced disease to take part and remain in a drug trial? A qualitative study. *Trials*, *21*(1), 215. <https://doi.org/10.1186/s13063-020-4129-2>
3. Majid T., Heath N., Kim J., West S.L., & dosReis S. (2021). Integrating the patient voice into pharmacoepidemiology research on the benefits and harms of medication. *Journal of Public Health (Germany)*, *(Majid, Kim, dosReis) Department of Pharmaceutical Health Services Research, University of Maryland School of Pharmacy, 220 Arch Street, Baltimore, MD 21201, United States*. <https://doi.org/10.1007/s10389-021-01477-2>
4. Michaels, D. L., Lamberti, M. J., Pena, Y., Kunz, B. L., & Getz, K. (2019). Assessing Biopharmaceutical Company Experience with Patient-centric Initiatives. *Clinical Therapeutics*, *41*(8), 1427–1438. <https://doi.org/10.1016/j.clinthera.2019.07.018>
5. Mollan S.P., Hemmings K., Herd C.P., Denton A., Williamson S., & Sinclair A.J. (2018). What are the research priorities for idiopathic intracranial hypertension? A priority setting partnership between patients and healthcare professionals. *Cephalalgia*, *38*(Supplement 1), 156–157. <https://doi.org/10.1177/0333102418801648>
6. Selig, W., Banks, I., Davis, A., DeCotiis, G., Hohman, R., & Schlager, L. (2019). Incorporating Patient Advocates in Oncology Clinical Development: Lessons Learned From a Novel Pilot Program. *Therapeutic Innovation & Regulatory Science*, *53*(3), 349–353. <https://doi.org/10.1177/2168479018790533>
